# Femtosecond‐Laser Preparation of Hydrogel with Micro/Nano‐Structures and their Biomedical Applications

**DOI:** 10.1002/smsc.202400400

**Published:** 2025-02-06

**Authors:** Jingyun Ma, Jinchi Wu, Zheli Lin, Jin Wang, Wenhao Yao, Yi Zhang, Xinquan Zhang, Limin Zhu, Yoshio Hayasaki, Honghao Zhang

**Affiliations:** ^1^ Ningbo Institute of Innovation for Combined Medicine and Engineering The Affiliated Lihuili Hospital of Ningbo University Ningbo 315000 China; ^2^ School of Mechanical Engineering Shanghai JiaoTong University Shanghai 200000 China; ^3^ School of Mechanical and Automotive Engineering Qingdao University of Technology Qingdao 266520 China; ^4^ Department of Otolaryngology Head and Neck Surgery Shanghai General Hospital Shanghai Jiao Tong University School of Medicine Shanghai 200000 China; ^5^ Laboratory of Laser and Medical Innovation Application (LLMIA) Gongli Hospital of Shanghai Pudong New Area Shanghai 200000 China; ^6^ Center for Optical Research and Education (CORE) Utsunomiya University Utsunomiya 321‐8505 Japan

**Keywords:** biomedical, femtosecond lasers, hydrogels, micro/nano‐structure

## Abstract

Hydrogels with micro/nano‐structures precisely prepared by femtosecond‐laser processing technology are biomaterials that mimic the natural extracellular matrix and can achieve the precise regulation of biological properties, such as cell behavior and structural–mechanical properties. They also play a crucial role in biomedical fields such as regenerative medicine, bionanostructural construction, drug transport, and particle manipulation. This study summarizes the central role of the nonlinear absorption property of femtosecond lasers in the preparation of hydrogel micro/nano‐structures. Based on the electron‐dynamics model and electron‐density flood theory, the two main processing types of the femtosecond laser‐processed hydrogel, additive and subtractive manufacturing, are discussed in depth. The modification mechanisms such as the photon–electron energy‐field conversion, multiphoton absorption effect, and bubble‐driven bio‐link molding are analyzed. Additionally, the stimulus‐response diversification and functional biomimicry properties of the structural hydrogels under the influences of different laser action modes and hydrogel composition ratios are investigated for their biomedical applications, such as microactuators, drug delivery, microscaffolds, and the in vitro simulation of vascular networks. On this basis, the advantages and limitations of the current technology are summarized and a reasonable prediction for future research on the law of action of the femtosecond‐laser preparation of hydrogel micro/nano‐structures is made.

## Graphical abstract

The review focuses on the femtosecond‐laser preparation of hydrogel micro/nano‐structures and their biomedical applications. It expounds the preparation principles, methods, biomedical applications, discusses the advantages and limitations, and looks forward to future development.

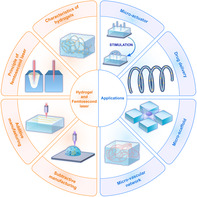

## 1 Introduction

Hydrogels are 3D polymer mesh structures formed by crosslinking. They exhibit full water‐absorbing expansion of the polymer network, belonging to the category of highly swollen fluid‐like solids. Hydrogels are water‐swollen, crosslinked hydrophilic polymers.^[^ ^1^ ^]^ Hydrogels exhibit a diverse range of forms and distinct properties and functions, and they typically possess the following characteristics. 1) Hydrogels have the capacity to retain substantial quantities of water molecules or biological fluids, with water content reaching up to 99%. This is why hydrogels are also called hydrophilic gels, and this characteristic allows them to mimic natural active tissues.^[^ ^2^ ^]^ 2) The structure of hydrogels closely resembles the extracellular matrix (ECM), a complex macromolecular network, enabling them to readily adapt to biological environments and facilitate the formation of biomimetic structures.^[^ ^3^ ^]^ 3) Hydrogels possess a loose, porous structure in which the pore size can be controlled to enable the diffusion of small molecules and even biological particles. These porous networks, which are composed of loose structures, can simulate the transport of nutrients, oxygen, and biologically active substances between various parts of different organs and tissues. Additionally, they assist in eliminating metabolic waste, such as acids and carbon dioxide, to maintain the internal homeostasis of the body. Without an interconnected microtubule network, the tissues cannot survive independently. 4) By precisely designing hydrogel polymer networks and controlling their structures at the molecular level, hydrogels with specialized properties such as responsiveness to stimuli such as temperature, pressure, potential of hydrogen (pH), and electric and magnetic fields can be achieved.^[^ ^4^ ^]^ The unique properties of hydrogels enable them to serve as reliable delivery vehicles for the targeted delivery of drugs, proteins, and peptides to specific sites. Furthermore, the ability to design and process these hydrogels in a stimulus‐responsive manner yields a diverse array of functional biomedical structures, including actuators, microfluidic valves, and various other applications. The adaptability and responsiveness of hydrogels render them invaluable in the biomedical field.^[^ ^1^ ^]^

Various types of hydrogels are required to achieve optimal structural properties for the diverse biomedical applications mentioned above. In this study, hydrogels are primarily categorized based on their sources and crosslinking forms. Hydrogels are classified as natural, synthetic, hybrid, or semi‐synthetic according to their source.^[^ ^2^, ^5^, ^6^, ^7^ ^]^ Natural hydrogels, such as chitosan, alginate, collagen, gelatin, and other natural sources utilized to fabricate hydrogel biomedical materials, exhibit excellent biocompatibility, degradability, and adhesion properties. However, natural hydrogels also possess disadvantages, primarily because of their inferior mechanical properties and lower stability compared to their synthetic alternatives.^[^ ^8^ ^]^ Synthetic hydrogels are composed of synthetic hydrophilic macromolecules that are physically or chemically crosslinked. Synthetic hydrophilic macromolecules include polyacrylic acid and its derivatives,^[^ ^9^ ^]^ polyvinyl alcohol,^[^ ^10^ ^]^ poly‐oxyethylene,^[^ ^11^ ^]^ and polyacrylamide.^[^ ^12^ ^]^ Their physical properties can be easily adapted for different applications.^[^ ^13^ ^]^ However, synthetic hydrogels also have disadvantages, such as relatively poor biocompatibility, possible photoinitiator toxicity, poor degradability, and loss of mechanical properties during degradation. Depending on the form of crosslinking, hydrogels are categorized as physical (reversible) or chemical (permanent) gels.^[^ ^14^ ^]^ Physical gelation is the most prevalent method for fabricating hydrogels, relying on molecular entanglement and other factors such as hydrophobic interactions or ionic/hydrogen bonding to unite networks and form hydrogel structures. The salient advantages of physical crosslinking are its biomedical safety, which stems from the absence of chemical crosslinking agents, and its self‐repairing and injectable properties at room temperature.^[^ ^15^ ^]^ Some researchers have studied physical gel.^[^ ^16^, ^17^, ^18^, ^19^, ^20^ ^]^ The hydrogels are self‐crosslinked through ionic interactions, and the resulting hydrogels have good cytocompatibility, good mechanical properties, and a dual healing ability.^[^ ^21^ ^]^ Chemical gels are 3D reticulated polymer hydrogels formed by crosslinking via strong chemical bonds. Compared to physically crosslinked hydrogels, chemically crosslinked hydrogels often exhibit greater stability under physiological conditions, superior mechanical properties, and tunable‐degradation behaviors.

In biomedicine and other fields, hydrogel materials are often used to simulate the in vivo microenvironment in vitro, including the study of the correlation between cell shape and cell function in a 3D environment and the study of the mechanism of cell‐substrate interaction, which is typically realized using fabricated high‐precision 3D micro/nano‐structures. Currently, numerous methods are available for preparing hydrogel micro/nano‐structures, including photolithography,^[^ ^22^ ^]^ wet spinning,^[^ ^23^ ^]^ 3D bioprinting,^[^ ^24^, ^25^ ^]^ and magnetic‐template methods. However, existing fabrication methods are limited by low processing precision, uncontrollable defects, and other challenges, which hinder the progress of related research.

The femtosecond‐laser processing method is different from the traditional hydrogel fabrication method. The hydrogel micro/nano‐structure fabricated by femtosecond‐laser processing technology has many advantages, such as controllable 3D morphology and high processing resolution, which have broad application prospects in biomedical fields such as bioprinting and tissue engineering.^[^ ^26^, ^27^, ^28^, ^29^, ^30^ ^]^ Lasers offer advantages such as high brightness, directivity, monochromaticity, and coherence. As a noncontact processing method, it can be processed on the surface of or inside the material in air, liquid, or vacuum environments. Femtosecond lasers are characterized by ultrashort pulse widths (several femtoseconds to hundreds of femtoseconds, 1 fs = 10^−15 ^s), ultrahigh peak power (up to a million billion watts), and accurate multiphoton absorption thresholds; thus, they can realize high‐precision laser processing in a short period, with very small spatial dimensions and under extreme physical conditions. Therefore, they can be used to realize high‐precision processing of complex micro/nano‐structures. Femtosecond lasers have advantages that are incomparable with other long‐pulse lasers. 1) They exhibit high precision, quality and spatial resolution. Femtosecond lasers can be focused to a very small size. The two‐photon absorption probability is directly proportional to the square of the laser power; thus, the photopolymerization reaction only occurs in the region of very high photon density at the center of the laser focal point, which breaks through the limit of the optical diffraction limit, and has an ultra‐high resolution.^[^ ^31^ ^]^ 2) A wide range of materials can be processed, from metals to nonmetals, biological‐cell tissues, and even intracellular mitochondria.^[^ ^32^ ^]^ Due to the ultra‐high peak power of femtosecond lasers, they can exceed the optical‐excitation threshold of a material. Theoretically, they can be processed on many types of materials. 3) Femtosecond lasers exhibit low energy consumption, no thermal fusion zone, and “cold processing.” Due to the ultra‐short pulse width of the femtosecond laser, the time of the photon and material is considerably smaller than the time of the lattice thermal conduction. Moreover, the energy is only diffused locally near the focal point; therefore, the thermal effect is low. The local area is near the focus of the diffusion of energy only; thus, the thermal effect is low, reflecting the characteristics of “cold processing”. 4) Femtosecond lasers are used in 3D processing and have no special requirements for the environment, Femtosecond laser and material interactions primarily occur in the laser focus, usually combined with a high‐magnification objective lens and 3D motion platform. The laser is positioned in the target processing area through point‐by‐point scanning to realize the fine processing of any 3D shape. In summary, femtosecond lasers are suitable for processing a variety of hydrogel materials to a certain extent, compensating for the shortcomings of traditional processing methods. Femtosecond‐laser technology has also been applied to the fabrication of micro/nano‐structured hydrogels.

**Figure 1:**
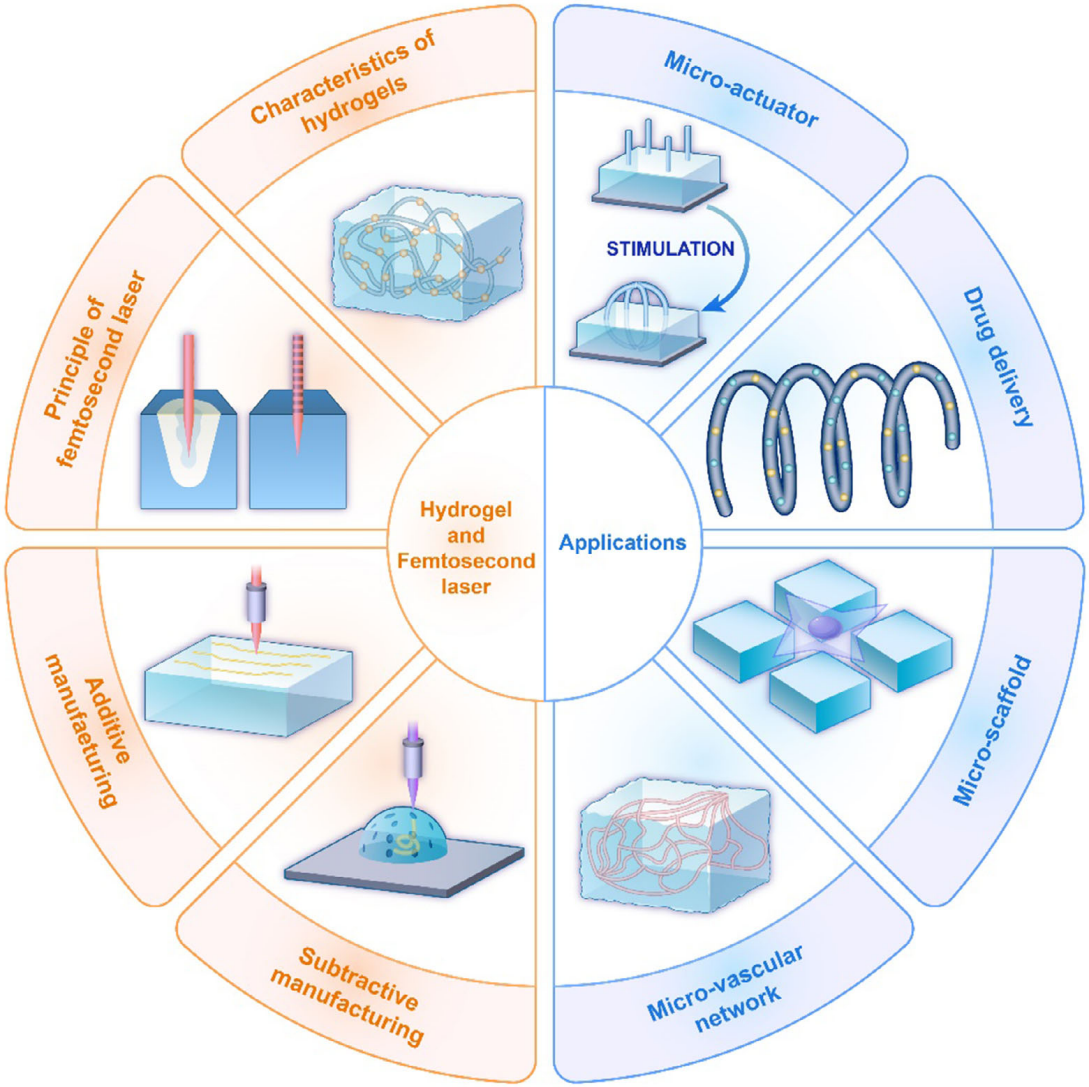
Femtosecond laser fabrication of hydrogel micro/nano‐structures and their biomedical applications.

This article presents a comprehensive overview of the principles of the femtosecond‐laser processing of hydrogels, emphasizing the significant research advancements and applications of biomedically relevant femtosecond laser‐induced hydrogel micro/nano‐structures in recent years, as illustrated in **Figure** **1**. Following the femtosecond‐laser fabrication of micro/nano‐structured hydrogels, we investigate the properties and prevalent classification methods of hydrogels, merits of femtosecond‐laser processing, and fundamental principles of preparing hydrogel micro/nano‐structures. Furthermore, we explore technical applications and usage scenarios of the fabricated hydrogels. Drawing from these insights, we discuss and forecast the challenges and development trends in this technology for hydrogel processing.

## 2 Principles and Techniques of Femtosecond‐Laser Fabrication of Hydrogel Micro/Nano‐Structures

**Figure 2:**
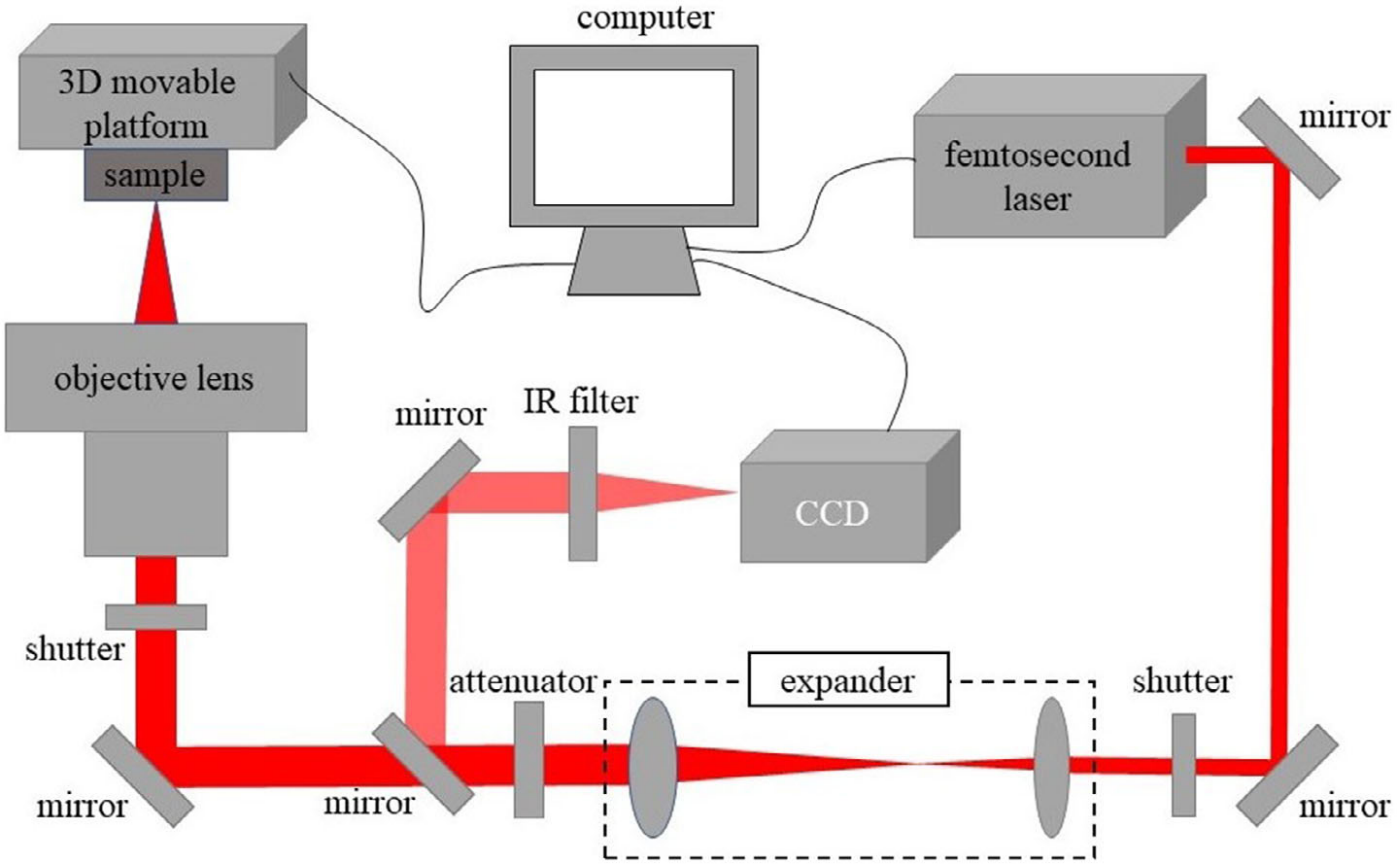
Schematic of femtosecond‐laser processing system.

The femtosecond‐laser fabrication of hydrogel micro/nano‐structures uses a femtosecond‐laser processing system to modify or manipulate a hydrogel with high precision to obtain high‐precision 3D hydrogel micro/nano‐structures and form a complete quantitative processing technology. The femtosecond‐laser processing system is mainly composed of a femtosecond laser, beam‐shaping module, 3D mobile platform, software‐control module, and other modules; its basic structure is shown in **Figure** **2**. The femtosecond‐laser beam passes through the laser‐beam expander and is collimated and parallelized to expand the beam. The attenuator is used to control the energy of the beam, which is then divided into two beams by a beam splitter, where one beam is intercepted and used to monitor the quality of the beam, and the other beam is introduced to the large‐numerical‐aperture (NA) focusing objective after a limited number of reflections. The beam is finally focused on the surface of the material to be processed or sent to the interior. A 3D shifting stage is used to carry the hydrogel material to be processed to ensure precise movement and accurate execution of the predefined 3D scanning paths. Femtosecond‐laser control software can control the laser power, scanning speed, open time, pulse frequency, and other important processing parameters to realize precise control of the laser energy at the focal point, improve the processing resolution, and efficiently form 3D micro/nano‐structures. According to the different technical principles of the femtosecond‐laser preparation of micro/nano‐structured hydrogels, this method can be divided into two major types: subtractive and additive processing. A detailed introduction to the technical principles of the two processing methods is presented in Figure 2.

### 2.1 Femtosecond‐Laser Material‐Reduction Processing of Hydrogels

Subtractive machining is a classic machining and manufacturing method that removes excess raw materials by precisely controlling the material‐removal process to create parts or products with specific shapes and specifications. Subtractive machining removes the material in its entirety and allows machining to be completed at room temperature, thereby avoiding problems with dimensional accuracy changes, such as shrinkage and expansion of the material due to changes in temperature and humidity. This method offers advantages such as good uniformity of the product structure, high material utilization, higher machining efficiency, and broader material compatibility. Hydrogel materials are typically colorless and transparent. The tight focusing capability and nonlinear absorption properties of femtosecond lasers in localized regions enable the ionization of transparent materials within a precisely defined heat‐affected zone. This facilitates highly accurate localized material removal at the micrometer and nanometer scales, thereby enabling femtosecond laser‐enabled material‐reduction processing. During processing, the material initially transforms into plasma and is subsequently removed because the laser‐plasma interaction results in material vaporization. The material‐removal process can be divided into two stages. First, material molecules undergo ionization owing to bombardment by a high‐energy particle beam. This ionization continues to generate free electrons, which heat and increase their kinetic energy. When the energy absorbed by the electrons exceeds the Fermi energy, charge separation occurs, and the electrons are pulled out of the material ions to form plasma. Macroscopically, this manifests as the laser ablation of the material, leading to plasma production, which completes the material‐removal process.

Photon–electron interactions during femtosecond laser–matter interactions dominate the entire nonequilibrium and nonlinear laser‐fabrication process, including the electron absorption of laser energy, electron‐to‐lattice energy transfer, plasma generation and phase transitions, and material modification. Theoretical models have been developed to explain the absorption of laser energy by electrons by revealing a portion of the ultrafast laser–material interactions. For time‐dependent electron‐dynamics models,^[^ ^33^ ^]^ based on time‐dependent density flooding theory, the time‐varying electron density is determined by solving a set of noninteracting Kohn–Sham equations using the electron density as a fundamental variable. The optical field is regarded as a spatially homogeneous exo‐vector potential that varies with time. The time‐varying Kohn‐Sham equations describing the electron motion are as follows

(1) $$ih\frac{\partial }{\partial t}\psi \left(\vec{r},t\right)=H_{\text{KS}}\left(\vec{r},t\right)\psi \left(\vec{r},t\right)$$

(2) $$n\left(\vec{r},t\right)=\sum_{i}{\left|\psi \left(\vec{r},t\right)\right|}^{2}$$

where $n\left(\vec{r},t\right)$ is the electron density and $H_{\text{KS}}\left(\vec{r},t\right)$ is the Kohn–Sham Hamiltonian quantity, which is routinely separated as follows

(3) $$H_{\text{KS}}\left(\vec{r},t\right)=\frac{1}{2m}{\left(p+\frac{e}{c}A\left(t\right)\right)}^{2}+V_{\text{ion}}\left(\vec{r},t\right)+V_{\text{Hartree}}\left(\vec{r},t\right)+V_{\text{XC}}\left(\vec{r},t\right)$$

where *e* is the unit charge and *A*(*t*) is the time‐ and external electric‐field‐dependent spatially uniform vector potential. $V_{\text{ion}}\left(\vec{r},t\right)$ is the electron–ion potential, $V_{\text{Hartree}}\left(\vec{r},t\right)$ represents the classical electrostatic interaction between electrons, and $V_{\text{XC}}\left(\vec{r},t\right)$ is the exchange‐correlation potential.

The time‐dependent electron‐dynamics model provides insights into the interactions between femtosecond‐laser pulses and materials. According to the model: 1) energy absorption and wavelength: at the same power density, the average absorbed energy per electron is almost inversely proportional to the laser wavelength. This means that shorter wavelengths (higher energies) lead to more energy being absorbed by individual electrons. Furthermore, as the laser power density increases, the number of excited electrons and the absorbed energy increase significantly. 2) Energy absorption and pulse string: when a pulse string composed of two pulses is used, the average energy absorbed by individual electrons is lower than that of a single pulse. This indicates that a smaller portion of the energy is converted into the intrinsic excitation energy of the electrons, whereas the remainder is used for electron emission. This improves photon efficiency, as more energy is utilized for material modification rather than being wasted as heat. 3) Pulse string technology and thermal effects: the pulse‐string technology extends the energy‐absorption time and reduces the maximum temperature of transient electrons. This not only retains the advantages of ultrashort pulse lasers, such as precise material removal, but also mitigates issues related to thermal cycling, such as stress fractures that can occur during laser material processing. 4) Pulse string modulation and electron distribution: by employing pulse‐string modulation technology, the pulse string consisting of multiple pulses acts on regions where more electrons are excited to the conduction band. This suggests that areas with a higher electron density are more prone to the nonlinear absorption of laser light. Consequently, high‐precision material removal at the micrometer and nanometer scales can be achieved. In summary, the model highlights the importance of understanding how electrons interact with femtosecond‐laser pulses and how the pulse parameters can be optimized to achieve the desired material‐modification outcomes. By adjusting the wavelength, power density, and pulse structure, we can precisely control the energy absorption and subsequent material responses, thereby enabling advanced laser‐based manufacturing techniques.

**Figure 3:**
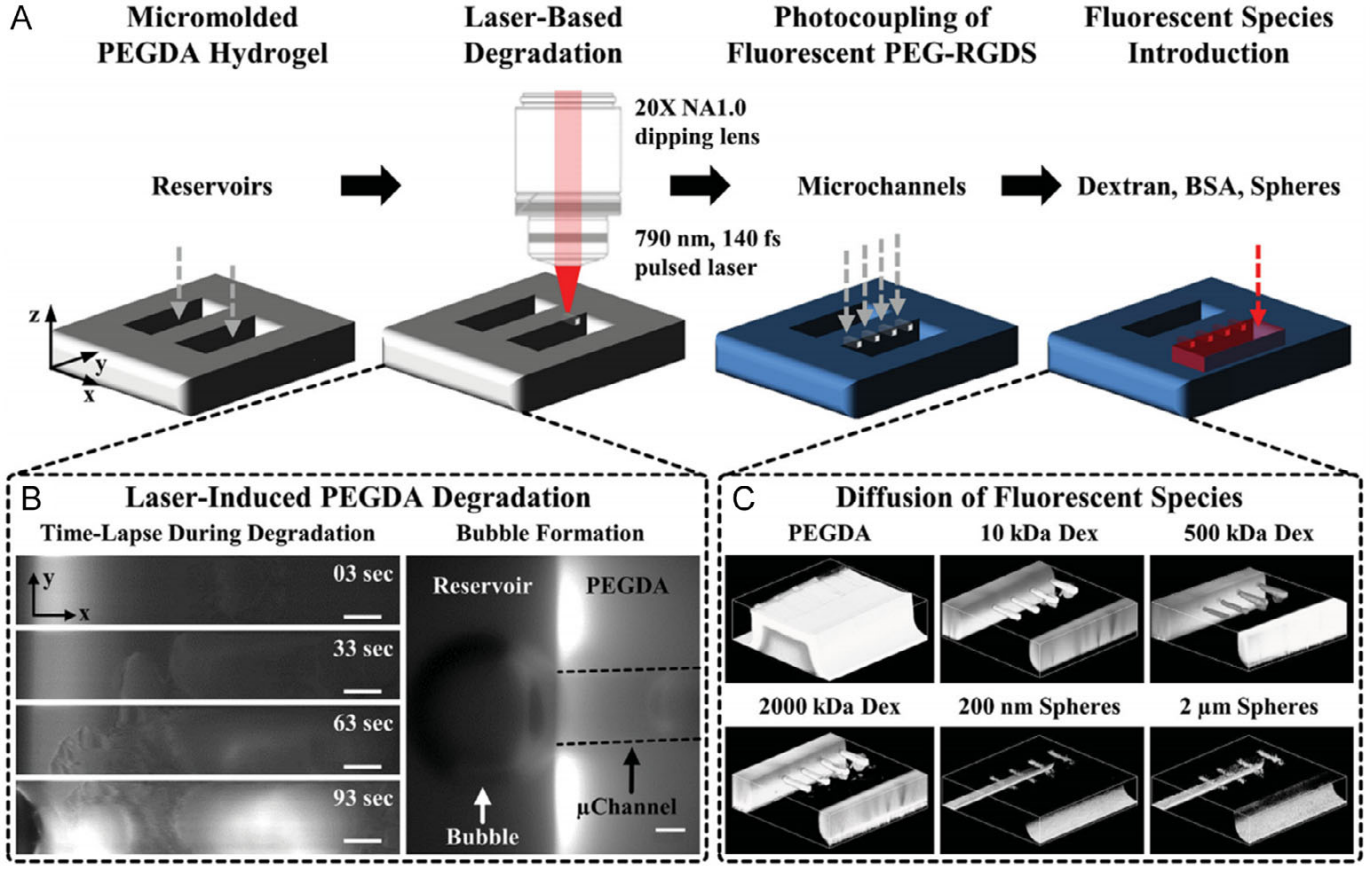
Preparation of microfluidic channels in PEGDA hydrogels. Reproduced with permission.^[^ ^34^ ^]^ Copyright 2016, Wiley‐VCH. A) A PEGDA hydrogel is photopolymerized to create reservoirs; B) time‐lapse images of PEGDA during laser‐induced degradation of a channel; and C) 3D renderings of a micromolded hydrogel and microchannels filled with fluorescent substances.

To overcome many of the limitations of existing microfluidic fabrication strategies, Heintz et al.^[^ ^34^ ^]^ combined laser‐based hydrogel degradation with image‐guided laser control to generate a complex, 3D, highly convoluted, and dense bionic microfluidic network embedded in polyethyleneglycol diacrylate (PEGDA) hydrogels. The network accurately mimics the in vivo vascular structure, as well as the transport between independent networks, opening up new avenues for the realization of vascularized tissue structures. Arakawa et al.^[^ ^35^ ^]^ developed a photodegradable‐material‐based approach to generate endothelialized 3D vascular networks in cell‐loaded hydrogel biomaterials with a fully customizable intraluminal channel structure for synthetic blood vessels. Takayama et al.^[^ ^36^ ^]^ developed a new approach for the production of endothelialized 3D vascular networks in hollow channels surrounded by gold nanoparticles, which were prepared in PEGDA, as shown in **Figure** **3**. Owing to the removal effect of femtosecond‐laser‐induced hydrogel degradation, the center of the channel is a cavity in PEGDA, and both the hydrogel and gold nanoparticles have good biocompatibility, and can be widely used in biomedical applications in the future.

### 2.2 Femtosecond‐Laser Additive Manufacturing of Hydrogels

Additive manufacturing technology is based on the principle of hierarchical manufacturing, that is, the use of the material layer‐by‐layer method of accumulation, directly to the digital‐model manufacturing of solid parts of a new manufacturing technology. Compared with traditional manufacturing technology, additive manufacturing technology provides a series of advantages, such as a high degree of freedom, the processing of complex 3D structures, and a wide range of processable materials.^[^ ^37^ ^]^ Femtosecond‐laser additive manufacturing of hydrogels is mainly classified into two methods: two‐photon polymerization (TPP) and laser‐assisted bioprinting.

#### 2.2.1 Femtosecond‐Laser‐Induced Photonic Polymerization

**Figure 4:**
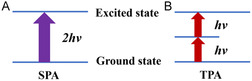
Schematic of the principles of A) single‐photon absorption and B) two‐photon absorption.

Femtosecond‐laser‐induced photopolymerization for additive manufacturing is primarily based on TPP and multiphoton polymerization (MPP) principles. In the femtosecond‐laser processing of hydrogels, the TPP effect is most common during electronic excitation. The TPP effect is a third‐order nonlinear optical effect in which a molecule simultaneously absorbs two photons with energies lower than that required for its electrons to jump to the lowest excited‐state energy level and jump from the ground state to the excited state.^[^ ^38^ ^]^ As shown in **Figure** **4**a, for the single‐photon excitation process, when the energy contained in a single photon is equal to the energy required for the electron to jump from the ground state to the excited state, the electron absorbs a photon and is excited. After a period of time, it returns to the ground state and releases fluorescence. When the wavelength of light increases to twice that of the single‐photon excitation process, the energy of a single photon is reduced to half, the derivation of which is shown in the following equation

(4) c=λν

(5) E=hν

where *c* is the speed of light, *λ* is the wavelength of light, *ν* is the frequency, and *E* is the energy of a single photon. When the light intensity is sufficiently strong, that is, the photon density is extremely high, the ground‐state electron can absorb two photons simultaneously and jump from the ground state to the excited state. The two‐photon excitation process is illustrated in Figure 4b.

**Figure 5:**
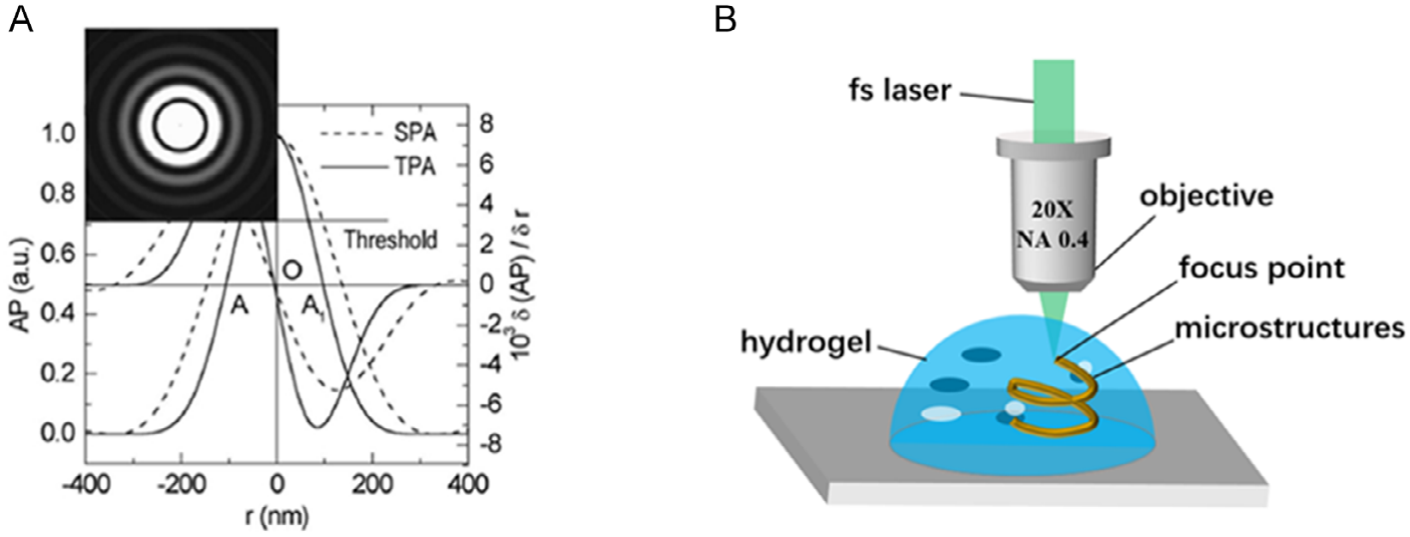
A) Schematic of achieving subdiffraction‐limited fabrication accuracy, where the absorption probabilities of single‐photon absorption and two‐photon absorption are represented by dashed and solid lines, respectively. The inset shows the diffraction pattern. Reproduced with permission.^[^ ^41^ ^]^ Copyright 2002, AIP. B) Schematic of femtosecond‐laser processing with two‐photon polymerization principle.

The extremely high density of photons generated by the femtosecond laser in a short time can generate sufficient excited‐state electrons, which will further cause the direct photoablation of the material.^[^ ^39^ ^]^ The decomposition of photosensitive substances in the excited state generates reactive substances such as free radicals or cations, which further trigger the monomer polymerization reaction, the so‐called TPP principle. The processing of hydrogels and many other transparent materials based on the TPP principle is able to achieve high resolution for three main reasons. 1) Focusing the laser beam through a lens with a high NA creates a focal spot with a very small diameter, concentrating a large number of photons in a very small focusing volume, which triggers a localized two‐photon absorption effect as well as a reduction in the machining size. 2) Due to the extremely short duration of the femtosecond pulse, no visible plasma is observed due to the accumulation of energy from high temperatures over time.^[^ ^39^, ^40^ ^]^ This effectively suppresses thermal diffusion and improves processing resolution. 3) The two‐photon absorption rate of the material and incident light intensity are nonlinearly related. Therefore, the laser‐light intensity can be controlled so that the power exceeds the TPP threshold at the laser focal point, but exceeds it to a much lesser extent than the spot area after focusing the lens, as shown in **Figure** **5**a.^[^ ^41^ ^]^ Using this method, the physical limitations of light‐field diffraction can be overcome, thereby realizing high‐resolution structural processing. In addition, the combination of this method with a high‐precision motion platform can accomplish the machining of reproducible freestanding continuous structures with a resolution of tens of nanometers, as shown in Figure 5b.

**Figure 6:**
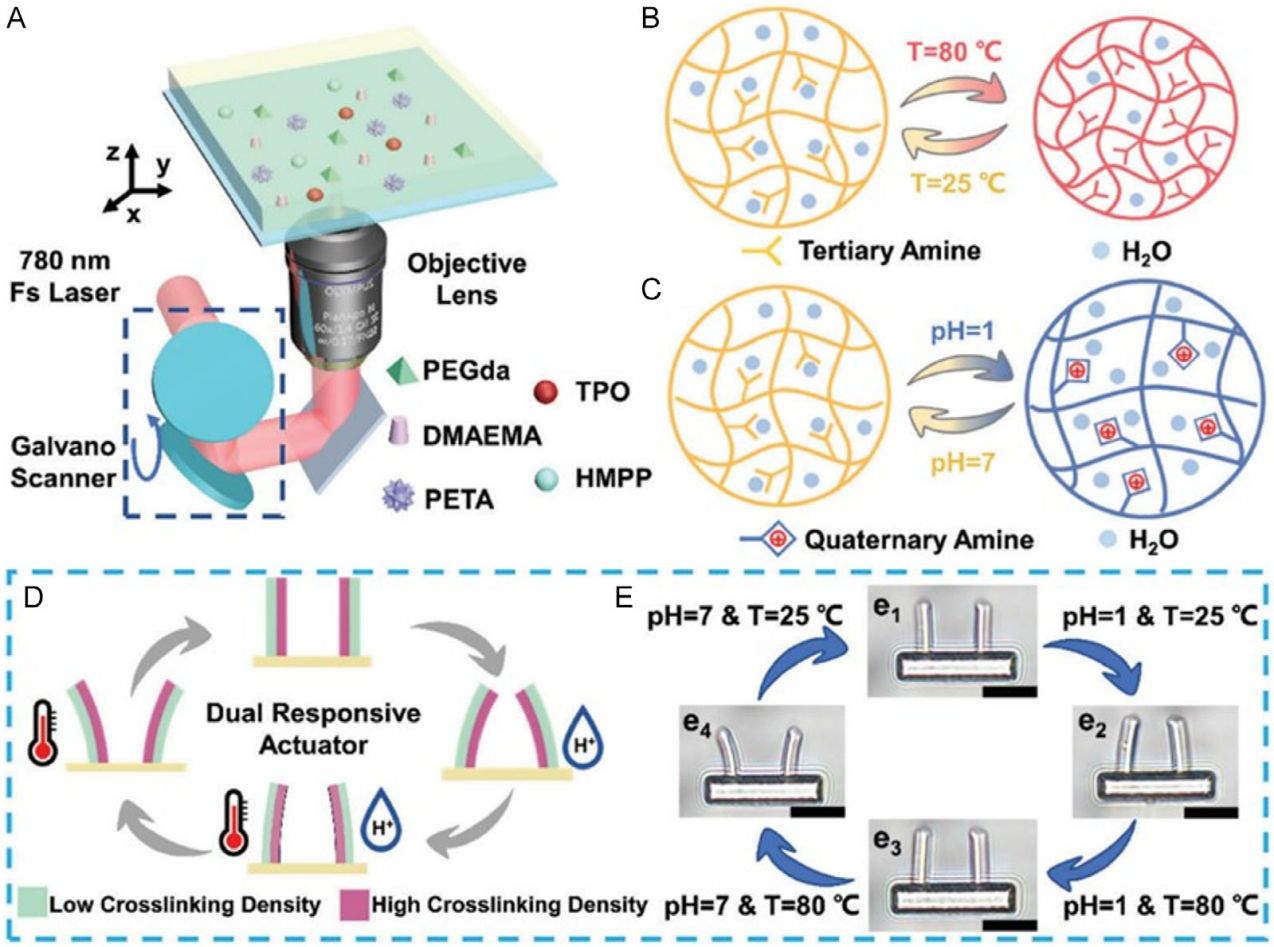
Schematic of femtosecond‐laser direct writing stimulus response micro/nano‐structure. Reproduced with permission.^[^ ^44^ ^]^ Copyright 2023, Wiley‐VCH. A) Schematic of fsLDW of dual‐stimuli responsive microstructure; B) states of polymer chains in hydrogel at 25 and 80 °C; C) states of polymer chains in hydrogel at different pH; D) dual‐stimuli cooperative response of hydrogel microactuator; and E) bright‐field images of hydrogel microactuators at various pH and temperature.

Ennis et al.^[^ ^42^ ^]^ proposed a phenylboronic‐acid‐based photoresist method compatible with TPP for fabricating micro/nano‐structures with a faster stimulus response. An easy‐to‐implement fabrication method for complex structures was developed by combining the flexibility of TPP with a more adaptable photoresist. Bin et al.^[^ ^43^ ^]^ proposed the use of an anionic cazolyl water‐soluble two‐photon initiator to build arbitrary 3D hydrogel structures. The water‐solubility, biocompatibility, and nonlinear absorption properties of the two‐photon initiator were further improved. Compared with previous initiators with iodide ions as anions, initiators with *p*‐toluenesulfonate as the anion have higher binding energies, larger two‐photon absorption cross‐sections, and two‐photon preparation resolutions, and they have great potential for application in biomedical fields. Wang et al.^[^ ^44^ ^]^ proposed a dual‐stimulus synergistic response hydrogel microactuator based on a femtosecond‐laser direct‐write asymmetric fabrication technique using a reactive hydrogel material with the functional monomer 2‐(dimethylamino)ethyl methacrylate to realize a dual‐stimulus response of the hydrogel micro/nano‐structure. The asymmetric hydrogel micro/nano‐structures show opposite bending directions when heated at high temperatures or in acidic solutions. They respond synergistically to changes in temperature and pH simultaneously, and are capable of independently grasping polystyrene microspheres (**Figure** **6**).

#### 2.2.2 Femtosecond‐Laser‐Assisted Bioprinting

**Figure 7:**
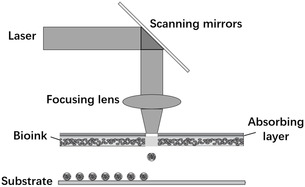
Laser‐assisted printing of biological cell deposition structures.

Femtosecond‐laser‐assisted bioprinting uses a droplet as the basic forming unit and is based on the principle of focused laser pulses, in which the expansion of bubbles drives biomaterials and cells off the substrate and deposits them onto the forming platform. As shown in **Figure** **7**, a standard laser‐assisted printing system consists of a pulsed laser, an absorber layer responsible for absorbing the laser energy (usually a glass layer overlaid with a gold or platinum layer), a bioink layer (a coated cell or a hybrid hydrogel prepared in a liquid environment), and the bottom‐most layer of the receiving substrate. The advantages of this method are that it can avoid direct contact between the bioink and processing device, and this noncontact manufacturing method will not cause mechanical shear damage to the cells. It can be used to print biomaterials with high viscosity (1–300 mPa s^−1^), it can deposit high‐density cells (up to a density of ≈108 cells mL^−1^), and it can realize precise control of each cell (accuracy of 30–100 μm). Its control‐laser frequency can reach more than 5 kHz, and it does not experience the problem of printing‐nozzle clogging compared with the traditional method.^[^ ^45^ ^]^ Additionally, it is applicable to a wider range of materials than inkjet printing. However, this method also has limitations: slow printing speed, complex and costly preparation of printing materials, lack of commercially available printing devices, time‐consuming coating of bioink on laser‐absorbent materials for each layer of printing, and difficulty in controlling the reproducibility of the resulting microdroplets.

**Figure 8:**
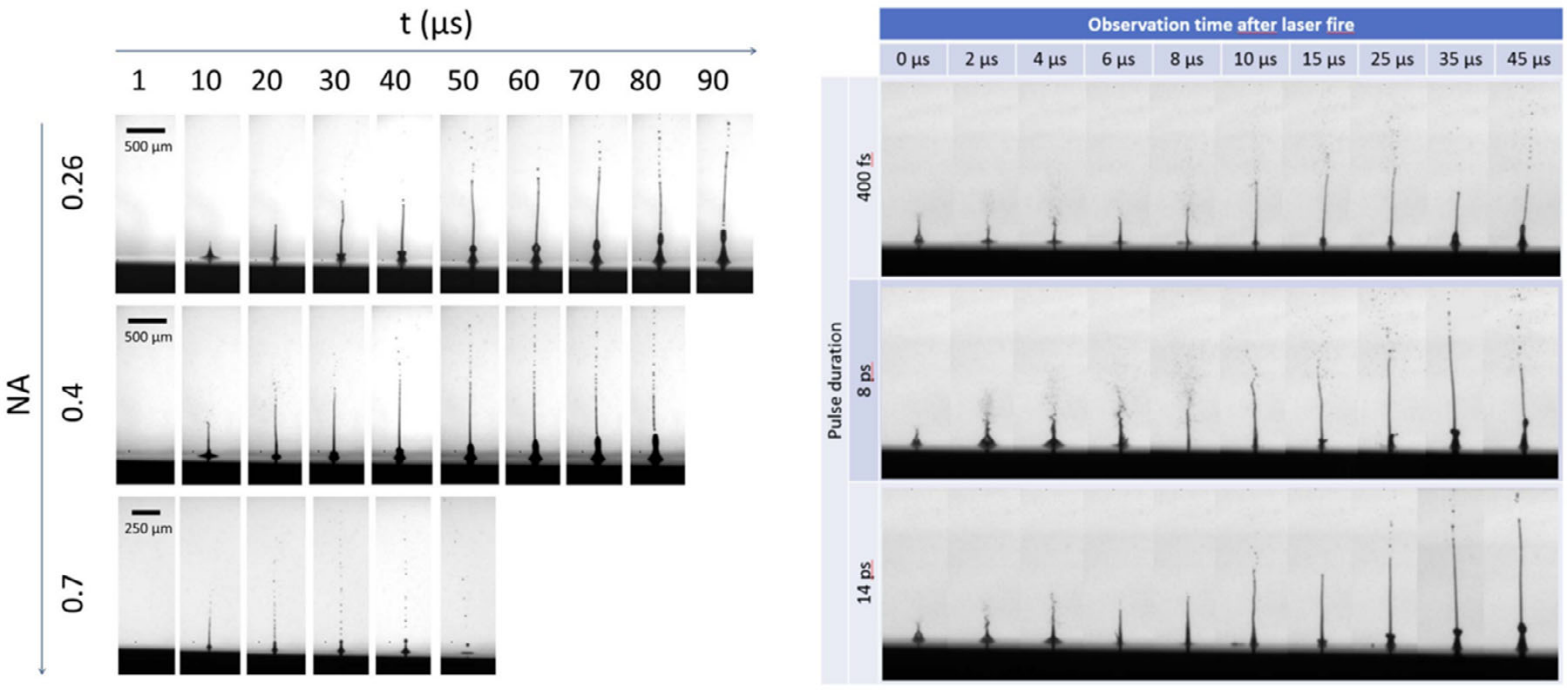
Effect of NA and pulse length on laser‐assisted bioprinting jets. Reproduced under the terms of the CC‐BY 4.0 license.^[^ ^47^ ^]^ Copyright 2017, The Authors. Published by Applied Optics.

Femtosecond lasers have higher peak pulse power compared with traditional long‐pulse lasers, which can realize the transfer of laser energy to the printing material through nonlinear absorption without an absorbing layer and directly generate bubbles to avoid the contamination of the absorbing layer. This has attracted extensive attention from researchers, and studies related to femtosecond laser‐assisted bioprinting have been conducted. Desrus et al.^[^ ^46^ ^]^ investigated the optimal parameters for the NA and laser‐focus‐position accuracy of a femtosecond‐laser bioprinting device without the use of a metal‐absorbing layer. The study utilized three bioinks with different surface‐tension and viscosity properties. Using time‐resolved imaging observations, the results showed that the NA of the objective lens and laser‐focusing position *z* had a greater effect on the maximum jet height of the bioink than on its own rheological properties. Moreover, the optimal NA value was determined as 0.4, and the *z*‐axis tolerance of the objective‐lens position for the generated jet was between the maximum jet heights of *h*_max_ and *h*_max_ −200 μm when the focusing position should be precisely controlled between 10 and 50 μm. Petit et al.^[^ ^47^ ^]^ investigated the properties of microjets induced in the absence of an absorbing layer using femtosecond and picosecond lasers. The effects of pulse length (0.4–12 ps) and energy (6–12 μJ) on jet height, diameter, velocity, volume, and shape were systematically investigated. The results showed that 400 fs pulses produce thin, stable, and spatially accurate jets with moderate initial velocities, which allow the design of complex and fine patterns of living cells. Longer pulses (>14 ps) produce jets that are unstable in time and energy, which are unsuitable for bioprinting applications. Moreover, 8 ps pulses produce jets that are larger in volume, have higher velocities, and propagate over longer distances, which can achieve a certain degree of print speed and print‐quality balance, covering many applications of laser‐generated microjet bioprinting (**Figure** **8**).

In summary, methods for the femtosecond‐laser preparation of hydrogel micro/nano‐structures can be generally divided into subtractive and additive processing. This section focuses on the role of the subtractive and additive processing mechanism and exploratory application research. It also outlines the advantages and shortcomings of each of the two methods and clarifies the direction of technological research to optimize the capability of femtosecond‐laser processing or provide innovative ideas for the future “additive/subtractive hybrid” manufacturing‐technology development by providing innovative ideas. The potential of the femtosecond‐laser fabrication of hydrogels for biomedical applications is described and illustrated in detail in the next section using specific research cases.

### 2.3 Comparison of Fabrication Methods

#### 2.3.1 Cost Comparison

Femtosecond‐laser systems used in material‐reduction processing and femtosecond‐laser‐induced photonic polymerization rely on advanced technologies and precise components, leading to high acquisition costs. Material‐reduction processing is relatively expensive because it requires a higher laser power. Generally, the higher the laser power, the more expensive the laser. As for femtosecond‐laser‐induced photonic polymerization, it is quite costly because it demands accurate power control and precise 3D displacement components. In contrast, 3D bioprinting equipment based on femtosecond lasers, with varying functional focuses, is comparatively less expensive but still more costly than non‐femtosecond‐laser 3D bioprinters. Technological advancements are gradually reducing these costs.

In terms of material costs, material‐reduction processing has a high utilization rate of hydrogels and imposes no restrictions on the types of hydrogels, so the costs are relatively low. Femtosecond‐laser‐induced photonic polymerization requires specialized photoinitiators and materials, which leads to relatively high costs. As for femtosecond‐laser‐based 3D bioprinting, the costs of bioinks vary depending on the selected materials, and the material costs mainly hinge on the bioinks chosen.

#### 2.3.2 Scalability and Precision Comparison

3D bioprinting utilizing femtosecond laser technology demonstrates an advantage in production scalability. By capitalizing on its layer‐by‐layer deposition principle and potential improvements, such as optimized print path design and increased printheads, it can rapidly fabricate 3D objects. Material‐reduction processing proves inefficient for large‐scale production scenarios. Its processing speed is influenced by factors such as laser power, scanning modality, and the volume of material to be removed, making it time‐consuming when processing large‐area or thick hydrogels. Femtosecond‐laser‐induced photonic polymerization is similarly constrained by its point‐by‐point or layer‐by‐layer polymerization approach. However, emerging holographic laser processing technologies are expected to enhance its scalability and speed.

Femtosecond‐laser‐induced photonic polymerization exhibits excellent performance in the scalability and precision of complex structures. It can fabricate nanoscale structures and overcome the diffraction limit. Under stable conditions, its precision is high, but it is affected by environmental fluctuations, and environmental control and real‐time monitoring are required to maintain precision. Material‐reduction processing has low precision in manufacturing complex structures. During long‐term operation, the precision will decline due to problems with the laser source and mechanical components, and regular calibration and maintenance are needed. Femtosecond‐laser 3D bioprinting can produce complex tissue‐like structures, but its precision at the micro/nano scale is limited. It is affected by factors such as bioink, nozzle, and laser energy, and it is difficult to maintain precision, requiring frequent inspection and adjustment of printing parameters and equipment.

#### 2.3.3 Practical Implementation Comparison

3D bioprinting with femtosecond laser technology primarily utilizes bioinks. In contrast, material‐reduction processing and femtosecond‐laser‐induced photonic polymerization are capable of handling a broader range of hydrogel materials. These techniques can adjust laser parameters based on the optical and physicochemical properties of the hydrogels, although they impose specific requirements on the optical properties of these materials.

3D bioprinting with femtosecond laser technology requires a stable environment and power supply. Material‐reduction processing and femtosecond‐laser‐induced photonic polymerization require more stringent environmental conditions, akin to those of a clean room, due to their high precision and sensitivity to environmental changes.

The postprocessing of 3D bioprinting with femtosecond laser technology primarily focuses on bioink curing and surface treatment to enhance structural strength and improve biocompatibility and cell affinity. Material‐reduction processing typically involves the relatively straightforward removal of residues. Femtosecond‐laser‐induced photonic polymerization necessitates the careful removal of unreacted monomers or small molecules due to its high precision.

The repeatability precision of material‐reduction processing is affected by factors such as the energy threshold for material removal and is lower than that of femtosecond‐laser‐induced photonic polymerization. Femtosecond‐laser‐induced photonic polymerization has good repeatability and can precisely fabricate complex nanoscale hydrogel structures. The repeatability of 3D bioprinting with femtosecond laser technology is influenced by multiple factors such as bioink and cell activity. It is relatively poor when fabricating biologically active complex structures, while a certain level of repeatability can be achieved through process optimization when fabricating simple or nonbiologically active hydrogel structures (**Table** **1**).

**Table 1:** Comparison table of femtosecond‐laser fabrication of hydrogel micro/nano‐structures.

Comparison aspects	Material reduction processing	Femtosecond‐laser‐induced photonic polymerization	3D bioprinting (with femtosecond laser)
Equipment acquisition cost	High, relying on laser power	High, due to precise components	Moderately high, with different functional focuses
Material cost	Low, high utilization rate	High, specialized photoinitiators	Variable bioink cost
Production efficiency scalability	Inefficient in large – scale. Speed affected by laser power, scanning strategy, material removal volume	Limited restricted by polymerization method. Potential improvement with holographic laser processing technologies	Efficient in quantity Speed depending on printhead, moving speed, bioink curing speed
Scalability of precision and structural complexity	Precision may decline due to laser source instability, mechanical moving part wear	Nanoscale excellent. Precision affected by temperature, humidity changes	Precision affected by bioink fluidity, nozzle clogging, laser energy fluctuations.
Material compatibility	Wide range	Many types, optical property‐related	Bioink of hydrogels‐ centered
Environmental requirements	Stable environment and power	Clean room environment	Stable, sensitive to environmental perturbations
Postprocessing requirements	Simple residue removal	Careful unreacted substance removal	Bioink curing and treatment
Repeatability	Affected by energy threshold, less precise than photonic polymerization	Excellent	Poor for bioactive complex

## 3 Biomedical Applications of Micro/Nano‐Structured Hydrogels Fabricated with Femtosecond‐Laser Technology

### 3.1 Microactuators

Microactuators based on 3D‐printed micro/nano‐structures in hydrogels with diverse functional groups exhibit automatic volume and shape responses to external stimuli. By adjusting the processing parameters, their response‐motion modes can be controlled, making them a type of 4D (3D + stimulus response) micro/nano‐structure. This innovation opens new avenues for the development of functional devices for applications such as drug delivery and biochemical analysis. Autonomous movement enabled by the stimulus response in these smart actuators lays a crucial technological foundation for various intelligent microdevices. The refined designs of these hydrogel micro/nano‐structures have wide applications in biomimetic structures, drug delivery, and particle manipulation.

**Figure 9:**
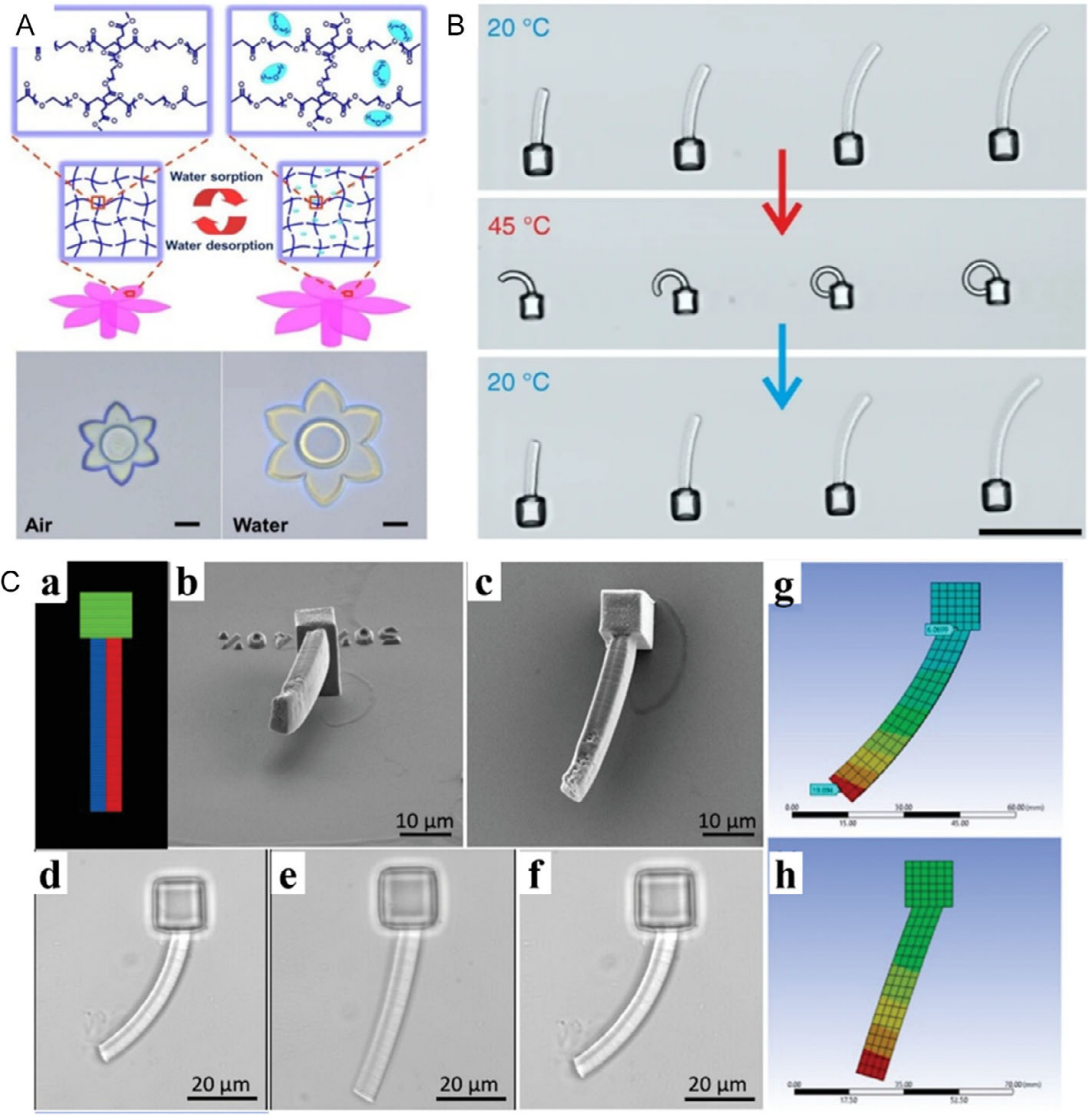
A) Humidity‐responsive flower‐shaped hydrogel micro/nano‐structure absorbs water and expands. Reproduced with permission.^[^ ^50^ ^]^ Copyright, 2018 Elsevier. B) Morphology of thermal‐responsive hydrogel cantilevers with varying lengths changes from 20 to 45 °C and back to 20 °C. Reproduced under the terms of the CC‐BY 4.0 license.^[^ ^51^ ^]^ Copyright 2019, The Authors. Published by Nature Communications. C) (a) Design of the bilayer beam, red denotes volume fabricated at 25 mW laser power, blue denotes volume fabricated 20 mW laser power, and green denotes the base of the beam fabricated at 30 mW; (b) SEM image of beam structure at 60°; (c) top‐down SEM image of beam structure; (d) optical microscope image of beam bending while hydrated in PBS; (e) optical microscope image of beam straightening while in 5 mm fructose solution; (f) optical microscope image of beam bending when the hydration media is reverted to PBS; (g) FEA models of beam in PBS solution; (h) FEA models of beam in 5 mm fructose.^[^ ^42^ ^]^ Reproduced under the terms of the CC‐BY 4.0 license.^[^ ^42^ ^]^ Copyright 2023, The Authors. Published by Advanced Functional Materials.

Xiong et al.^[^ ^48^ ^]^ investigated the macroscopic ion‐responsive properties of a crosslinked poly (AAm‐AMPS) hydrogel. When a photopolymerized gel disk containing 20.25 mol% AMPS was immersed in water, it expanded from 0.92 to 1.56 cm along the disk diameter at an expansion rate of ≈70%. Compared with large‐scale hydrogel structures, the prepared hydrogel microcantilevers showed a shorter ion‐concentration response time (0.133 s), indicating the significance of 3D hydrogel micro/nano‐structures with stimulus‐responsive characteristics in biomedical applications. Lay et al.^[^ ^49^ ^]^ fabricated various controllable circular‐to‐polygonal and polygonal‐to‐circular deformable bovine serum albumin (BSA) micro/nano‐structures, guided by three empirical rules. These rules include the triangular frameworks essential for circular‐polygonal conversion involving arc‐angle/side transitions, directing spiky frameworks to polygon corners for angle/side‐to‐arc transitions, and controlling the number of angles/arcs created by the embedded framework count. These deformable micro/nano‐structures were further combined to construct a 2D array with reversible deformation capabilities, showing strong potential for microfluidic control applications. Lv et al.^[^ ^50^ ^]^ utilized 3D printing via femtosecond laser direct‐writing technology to fabricate PEGDA hydrogel micro/nano‐structures with humidity‐responsive properties, which enabled expansion and volume changes under humid conditions. The swelling properties of the PEGDA micro/nano‐structures could be controlled by adjusting the voxel crosslinking density during fabrication. After further optimization of the laser parameters and conditions, controllable hydrogel nano‐interconnected networks were produced, achieving the interconnectivity of discrete micro/nano‐structures. Additionally, bioinspired hydrogel micro/nano‐structures mimicking plant‐leaf stomatal opening and closing processes have been created, demonstrating good repeatability and durability over thousands of humidity cycles. These micro/nano‐structures have broad application potential in sensors, actuators, and soft robots. Hippler et al.^[^ ^51^ ^]^ employed laser two‐photon lithography (TPL) to create poly(N‐isopropylacrylamide)‐based 3D heterogeneous structures by controlling the local material‐structure variations in a single production step using grayscale lithography. This has led to the development of stimulus‐responsive self‐moving structures with significant amplitude responses to temperature changes, which are activated either through global water‐temperature alterations or local micro/nano‐structure illumination via laser focusing. Jin et al.^[^ ^52^ ^]^ developed a 4D laser‐writing technique for manufacturing reconfigurable micromechanics capable of 3D reconstruction based on the adjustable stimulus‐responsive characteristics of each high‐resolution region of the hydrogel, offering feedback control over laser parameters. The use of commercial acrylic acid and N‐isopropylacrylamide materials in experiments, which can be replaced by other free‐radical polymerizable monomers, advances the steady development of preparation methods for various stimulus‐responsive functional micro/nano‐structures. These micro/nano‐structures are poised to become vital materials for potential biomedical applications, such as smart intravascular stents and artificial heart valves. Ennis et al.^[^ ^42^ ^]^ used a novel benzylboronic‐acid‐based photoresist to create sugar‐responsive hydrogel micro/nano‐structures. Exposure to a fructose solution resulted in polymer‐network expansion due to the repulsion between the negatively charged boronate esters. Further investigations revealed that with increased laser exposure, the driving force for sugar‐responsive volume changes in the hydrogel decreased, and the trend based on laser dosage was proven to be reversible—after several cycling changes, no signs of fatigue were observed in the micro/nano‐structures. Therefore, by controlling the laser dosage, the driving response can be quantitatively regulated. This method is applicable for creating complex microdevices that exhibit directed responses to stimuli, such as those used in continuous blood‐glucose monitoring and dual‐material microforceps applications (**Figure** **9**).

Building on 4D hydrogel microactuators, continuous innovations in fabrication processes have further enhanced their performance and enriched their functional diversity. Deng et al.^[^ ^53^ ^]^ proposed an innovative femtosecond laser‐based 4D printing strategy for fabricating light‐driven intelligent micromachines with programmable 3D shape‐morphing capabilities. The approach utilizes a carbon nanotube‐doped N‐isopropylacrylamide (NIPAM) composite hydrogel (CNNC), which enhances light absorption, thermal conductivity, and mechanical modulus, addressing the trade‐off between stiffness and responsiveness in conventional stimuli‐responsive hydrogels. By employing femtosecond laser direct writing (FsLDW), the authors achieved a single‐step fabrication of heterogeneous micromechanical metamaterial structures with precise spatial and temporal control. Key achievements include the fabrication of light‐responsive microstructures such as micropillar cilia, unidirectional heart valves, and the world's smallest artificial beating heart (80 × 120 × 60 μm^3^), demonstrating rapid responsiveness (300–400 ms), low actuation thresholds, and tunable shape‐morphing dynamics. This work establishes a versatile platform for the development of multifunctional intelligent micromachines applicable to biomedicine, tissue engineering, and soft robotics. Wu et al.^[^ ^54^ ^]^ proposed a novel temperature‐regulated capillary force self‐assembly strategy for fabricating reversible and switchable chiral microstructures using 4D‐printed hydrogel micropillars. By leveraging the deformation differences in hydrogels with asymmetric cross‐linking densities, this method achieves bidirectional bending and precise control over microstructure chirality through temperature variation. The approach demonstrates rapid response times (<200 ms), high repeatability, and scalability, enabling the assembly of complex hierarchical chiral architectures with tunable optical and mechanical properties. These advances highlight the potential applications of smart hydrogel‐based microstructures in chiral photonics, optical sensing, and microfluidic systems. Tao et al.^[^ ^55^ ^]^ developed a fsLDW strategy to fabricate monolayer heterojunction interactive hydrogels (MNIHs) with high‐resolution and high‐freedom 4D shape reconfiguration capabilities. By incorporating N‐isopropylacrylamide (NIPAM), polyethylene glycol diacrylate (PEG‐DA), and triallyl isocyanurate (TAIC) into a hydrogel network, combined with a methylene blue‐based two‐photon polymerization process, the authors achieved sub‐micrometer precision and tunable mechanical properties. These MNIHs demonstrated complex programmable behaviors such as chiral torsion, reversible 2D‐to‐3D transformations, biomimetic gripping motions, and self‐repairing capabilities. Additionally, the structures exhibited high biocompatibility (>97% cell viability) and superior mechanical adaptability (force‐to‐weight ratio >1000 N mg^−1^). This work highlights the potential of fsLDW in designing dynamic, multifunctional hydrogel microstructures for applications in biomedicine, microfluidics, and adaptive photonics.

Microactuators are primarily applied in three areas: biomimetic structures, particle manipulation, and embedded components of microfluidic chip devices.

#### 3.1.1 Biomimetic Structures

**Figure 10:**
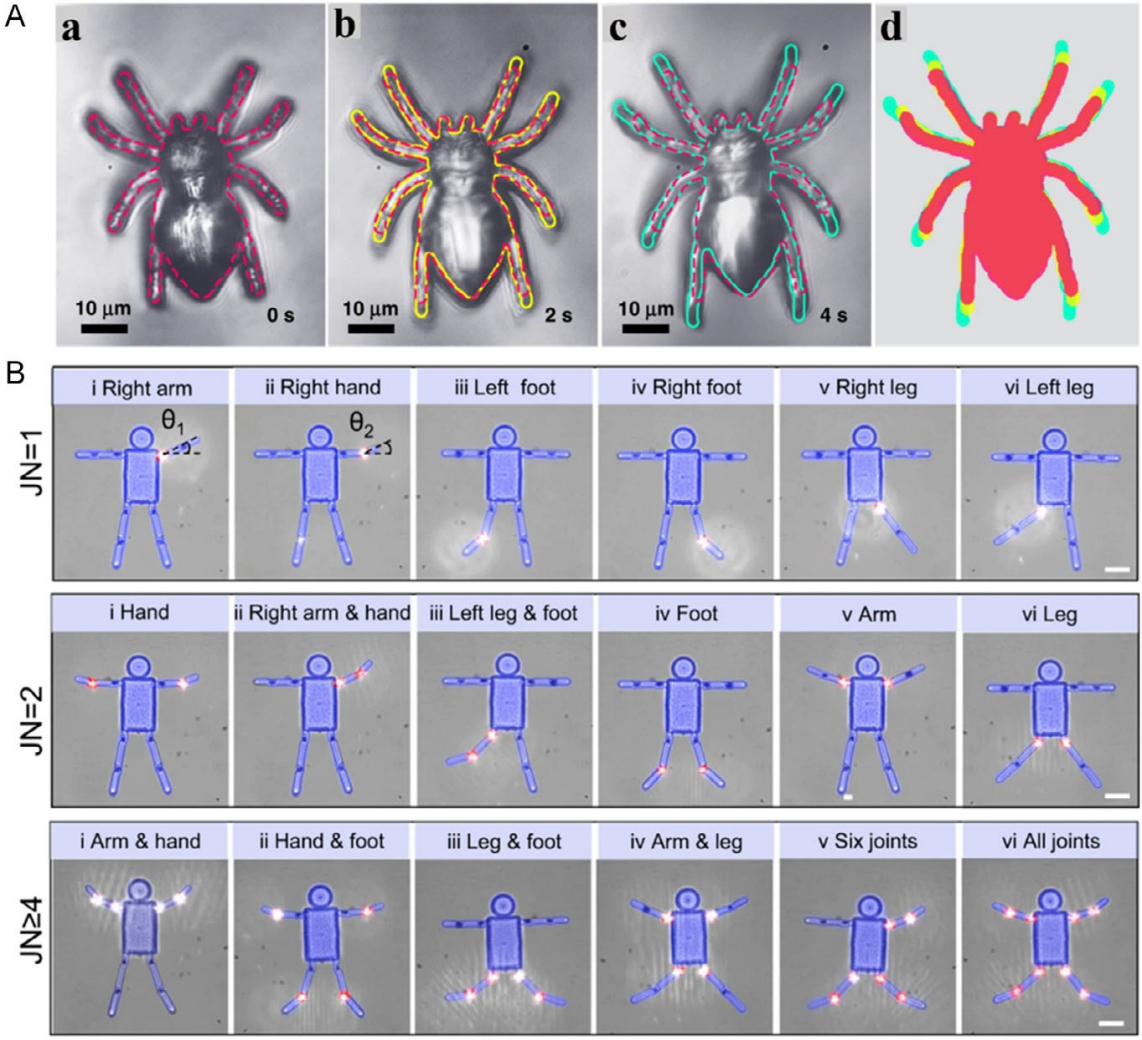
A) (a)–(c) The actuation of the microspider when the pH value was switched from 13 to 5. The profiles of the microspider in this actuation are marked in red, yellow, and green, respectively; (d) the superposition of the contour profiles at different time is provided for comparison. Reproduced under the terms of the CC‐BY 4.0 license.^[^ ^56^ ^]^ Copyright 2020, The Authors. Published by Nature Communications. B) Motions of biomimetic robots with single‐ (JN = 1), double‐ (JN = 2), and multijoint (JN >= 4) configurations. Reproduced under the terms of the CC‐BY 4.0 license.^[^ ^57^ ^]^ Copyright 2023, The Authors. Published by Nature Communications.

In the design and manufacturing processes of microbiology‐inspired robots, the creation of biomimetic microactuator structures capable of automatic responsive movements at the micro‐ and nanoscales has always faced significant technical challenges. However, the development of stimulus‐responsive hydrogel micro/nano‐structures offers new possibilities for addressing this technological hurdle. Ma et al.^[^ ^56^ ^]^ utilized fsLDW to simultaneously process a relatively rigid photoresist SU‐8 and pH‐responsive BSA at the submicron scale. Using SU‐8 as the skeleton and BSA as the muscle, they fabricated a pH‐responsive muscle–skeleton system. Through the dual‐photon polymerization processing characteristics, they programmed the internal networks of BSA and SU‐8 at the nanoscale, flexibly adjusting the elasticity of the muscle and stiffness of the skeleton. Building on this theory, they successfully integrated a pH‐responsive spider microrobot composed of this muscle–skeleton system (**Figure** **10**A), creating arm‐ and crab‐claw‐muscle systems. These systems exhibited rapid stimulus responses during testing, along with excellent durability and stability, enabling the capture and release of microtargets by controlling pH variations. Xin et al.^[^ ^57^ ^]^ prepared thermosensitive hydrogel microjoints with silver nanoparticles deposited on the surface using fsLDW. Multiple microjoints formed a microactuator with various modes of motion. The densely packed silver nanoparticles induced a strong photothermal conversion effect, resulting in a low driving power (10 mW) and short response time (30 ms) for the hydrogel microjoints. The silver nanoparticles absorbed photon energy and converted it into heat, achieving the precise driving of single‐ or multijoint coordinated responses. This capability enabled the microactuators to reconstruct various complex humanoid‐like movements (Figure 10B). In the future, intelligent stimulus‐responsive hydrogel microjoints may have broader applications in biomedical therapeutics. Inspired by the cooperative behavior of ant colonies, Ren et al.^[^ ^58^ ^]^ introduced a novel approach to create reconfigurable microbot collectives using femtosecond laser writing. The microbots are fabricated through a flexible TPP process, utilizing magnetic photoresist for the rigid body, thermo‐responsive hydrogel for flexible joints, and silver nanoparticles (Ag NPs) for photothermal actuation. These microbots, under the synergistic control of magnetic and light fields, can selectively and reversibly assemble into multiple configurations (e.g., 90 and 180° assemblies), enabling complex collective behaviors such as gap traversal, micro‐object manipulation, and adaptive cargo transport. The deformable hydrogel joints allow for stable assembly without continuous external stimuli, making the assembled structures highly robust. In addition to magnetic‐driven actuation, the light‐triggered photothermal effect of the Ag NPs induces rapid deformation of the joints, facilitating precise mandible movement. The study demonstrated the potential of these reconfigurable microbot collectives for applications in biomedical engineering, including drug delivery, with a proof‐of‐concept experiment showing significant cell inactivation in targeted regions. This work expands the functionality of microbot collectives by combining magnetic and optical control, providing new avenues for micromanipulation and targeted therapy.

#### 3.1.2 Particle Manipulation

Particle manipulation and selection are important in fields such as biomedicine and chemical analysis. Traditional methods encounter challenges at the micrometer scale, including low processing accuracy, difficulties in automation control, poor mechanical performance, and high manufacturing costs. Stimulus‐responsive hydrogel micro/nano‐structures, with their submicron high‐precision and dynamic responsive characteristics, offer new technological support for overcoming these obstacles.

**Figure 11:**
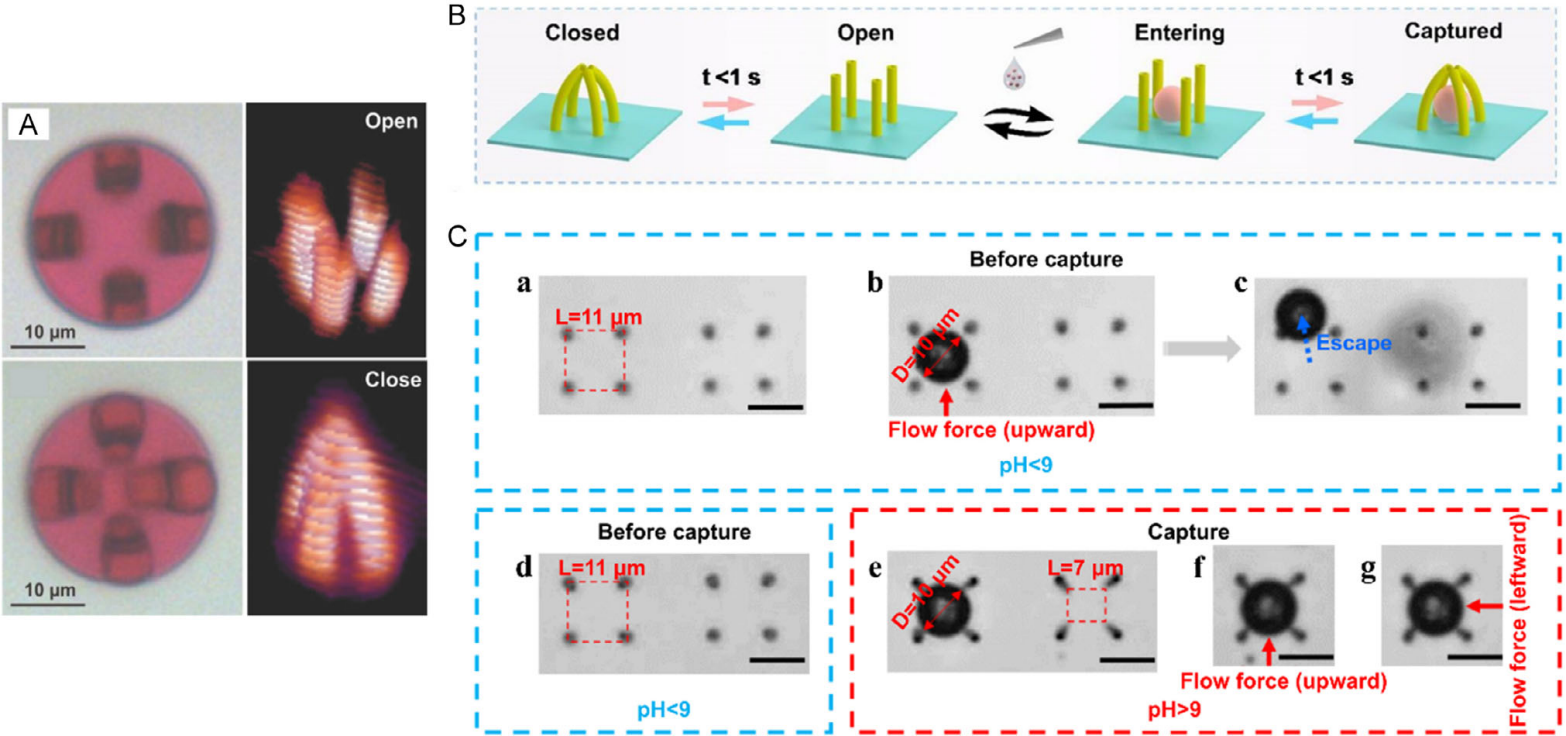
A) Open state and close state of pH‐responsive 3D microtraps. Reproduced with permission.^[^ ^59^ ^]^ Copyright 2014, Wiley‐VCH. B) Multifinger pH‐responsive microgrippers capture polystyrene microspheres within 1 s. Reproduced with permission.^[^ ^60^ ^]^ Copyright 2020, ACS. C) (a)–(c) PS microsphere (10 μm) can enter and overflow the open space with pitch of 11 μm, which was composed of four straight micropillars when pH < 9; (d) open space with pitch of 11 μm before capture; (e–g) a trapped microsphere that can withstand extra upward and leftward forces. Scale bars: 10 μm. Reproduced under the terms of the CC‐BY 4.0 license.^[^ ^61^ ^]^ Copyright 2021, The Authors. Published by International Journal of Extreme Manufacturing.

Particle manipulation is typically achieved based on the actuation principles of pH‐responsive microactuators, which are commonly composed of four to six specially designed crosslinked density microcolumns. These microcolumns can selectively bend upon pH changes, enabling the collective capture or release of particles. Lee et al.^[^ ^59^ ^]^ measured Young's modulus of a BSA hydrogel using atomic force microscopy and showed that increasing the laser‐writing distance along the *Z*‐axis from 100 to 500 nm decreased the modulus of the structure. By controlling the crosslinking density of the gel at the nanoscale through laser‐writing‐distance adjustments, the degree of expansion of the micro/nano‐structure could be quantitatively controlled with high precision. Using this segmented modulus approach, they designed stimulus‐induced chiral deformable micro/nano‐structures and fabricated 3D microtraps that could open and close according to the pH changes for particle trapping and release (**Figure** **11**A). Li et al.^[^ ^60^ ^]^ prepared pH‐responsive microactuators composed of hydrogel microtubes using density‐controllable femtosecond Bessel beams. By combining dynamic holographic processing and splicing methods, S‐shaped, C‐shaped, multistage microtube, and multivalve torsional chiral structures were achieved, thereby enhancing the deformation capability of the microactuators. They further utilized these structures to fabricate multifinger‐responsive microgrippers that successfully captured polystyrene microspheres and neural stem cells (Figure 11B). Lao et al.^[^ ^61^ ^]^ proposed a simple and flexible repetitive scanning strategy for manufacturing pH‐driven deformable Janus microcolumns. Unlike other strategies for constructing Janus structures, this strategy overcame processing limitations such as the need for different materials, varied process parameters, and multistep manufacturing processes. The repetitive scanning strategy involved scanning and processing one side of the Janus microcolumn structure followed by scanning and processing the other side. The structures formed by the initial scans scattered the laser beam during subsequent processing, resulting in a lower polymerization of the structures processed subsequently, leading to spontaneous heterogeneity in the microcolumns. Using this strategy, they created pH‐responsive Janus microactuators with a large bending angle of ≈31° and an extremely short response time of ≈0.2 s. In addition, they manufactured arrays of position‐ and curvature‐controlled Janus microactuators capable of capturing polystyrene microspheres (Figure 11C).

**Figure 12:**
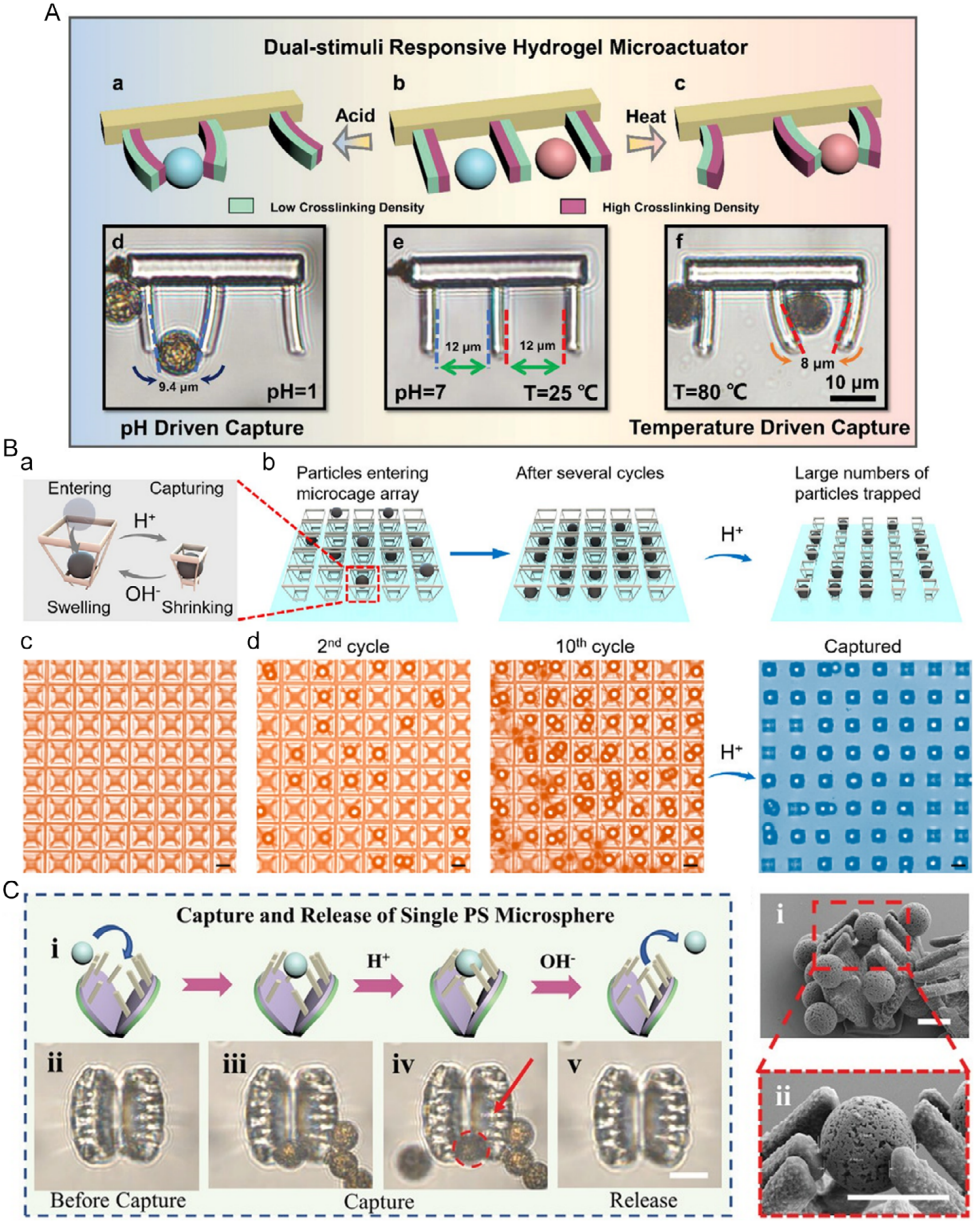
A) Morphology of temperature‐pH dual‐stimuli‐responsive microactuator changes with pH decreasing from 7 to 1 or temperature increasing from 25 to 80 °C. Reproduced with permission.^[^ ^44^ ^]^ Copyright 2023, Wiley‐VCH. B) pH‐responsive microcages for capturing large amounts of microparticles. (a) Illustration showing a single microcage swells and shrinks to capture a microparticle; (b) schematic diagram of the capturing process of the microcage array. After large amounts of particles enter the structures in several dropping cycles, HCl solution is added to trap particles inside the microcages; (c) optical images of the microcage array in its swelling state. The interval between each structure is 40.5 μm; (d) experimental diagram of the capturing process. The left two images show the microcage array after 2 and 10 dropping cycles. The right image shows the large numbers of particles trapped in the array after finally shrinking the structures and flushing away free particles. Reproduced with permission.^[^ ^63^ ^]^ Copyright 2022, ACS. C) pH‐responsive Venus‐flytrap hydrogel microactuator captures single PS microsphere when absorbing H^+^ and releases when absorbing OH^−^. Reproduced with permission.^[^ ^62^ ^]^ Copyright 2022, Wiley‐VCH.

Moreover, increasingly diverse microactuators are being developed for particle manipulation. Inspired by the Venus‐flytrap structure, Wang et al.^[^ ^62^ ^]^ fabricated stimulus‐responsive biomimetic asymmetric microactuators using a photoresist containing the pH‐responsive monomer 2‐(dimethylamino) ethyl methacrylate (DMAEMA) as the substrate. By adjusting the parameters of the femtosecond‐laser direct writing to control the local crosslinking density of the hydrogel, the responsive behavior of the hydrogel micro/nano‐structures can be designed accordingly. By utilizing asymmetrical deformation based on pH‐induced effects, micro/nano‐structures similar to Venus flytraps have been designed to capture single or multiple microspheres. Moreover, precise control of the release behavior of multiple microspheres was achieved using different release strategies (**Figure** **12**C). Zhang et al.^[^ ^63^ ^]^ overlaid the phase distributions of calculated holograms with different lenses based on holographic femtosecond multi‐focus technology, enabling arbitrary focal‐point positioning in 3D space with high precision and flexibility. A pH‐responsive microcage array manufactured using this processing method achieved the high‐performance capture of numerous particles (Figure 12B). Customized support arrays can serve as reliable analytical platforms for studying 3D cell behavior or evaluating drug efficacy, demonstrating strong application potential in the field of biomedicine. Wang et al.^[^ ^44^ ^]^ employed a controlled crosslinking density fsLDW asymmetric processing method to fabricate dual‐stimuli‐responsive hydrogel microactuators using the functional monomer DMAEMA. The tertiary amine group in DMAEMA acts as a strong hydrogen‐bond acceptor, forming hydrogen bonds with water at room temperature. As the temperature increases, the hydrogen bonds between the molecular chains and water break, leading to a decrease in the distance between the chains and polymer‐network contraction, yielding temperature‐responsive behavior. Simultaneously, the tertiary amine group is protonatable; in an acidic environment, protonation‐induced electrostatic repulsion increases the distance between the molecular chains, causing polymer network swelling, thereby exhibiting pH‐responsive behavior. By controlling the laser parameters, hydrogel microarms with controllable crosslinking densities were produced, capable of bending in opposite directions when exposed to high temperatures or acidic solutions, thereby enabling the independent grasping of polystyrene beads (Figure 12A). These dual‐stimuli‐responsive hydrogel microactuators have significant potential for applications in soft robotics and microscale object manipulation.

**Figure 13:**
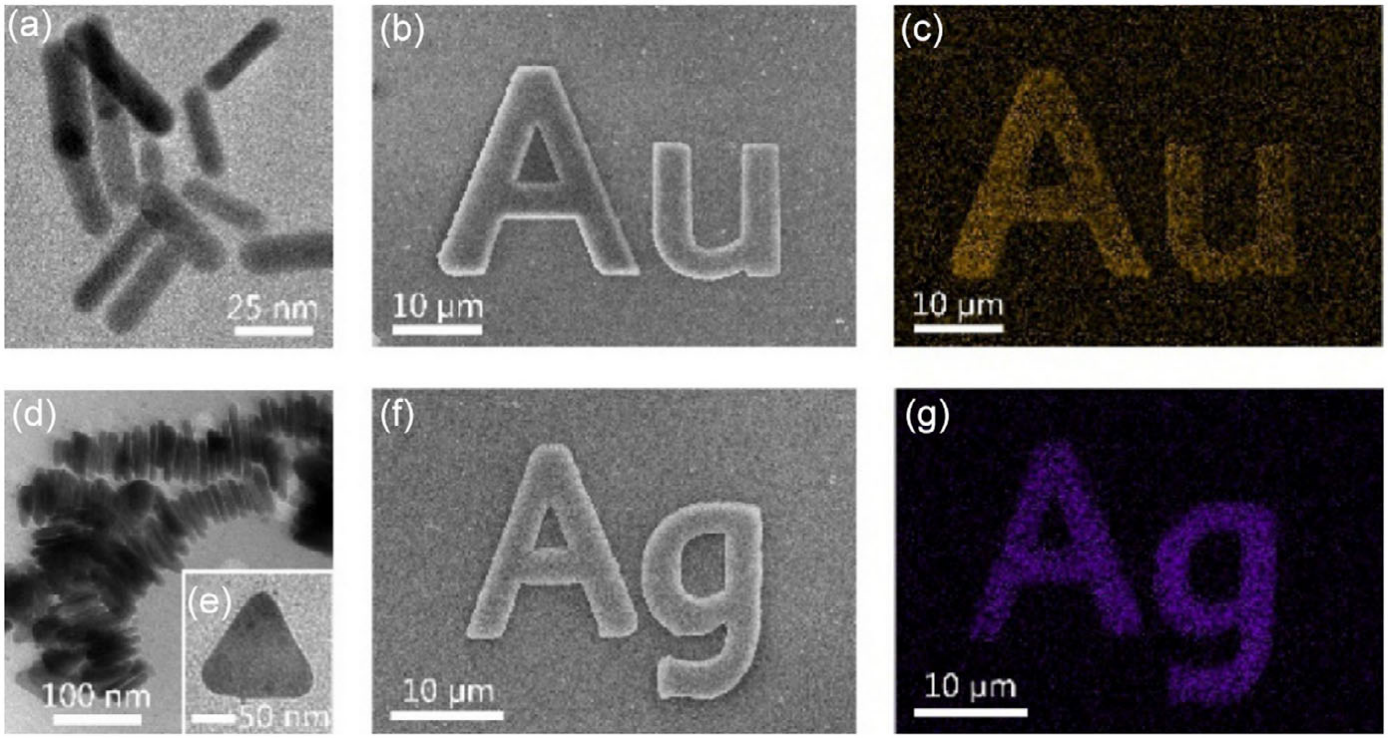
a–c) “Au” PAMPS pattern adsorbing positively charged gold‐nanoparticle ions and d–g) “Ag” PAMPS pattern adsorbing negatively charged silver nanoplates. Reproduced under the terms of the CC‐BY 4.0 license.^[^ ^64^ ^]^ Copyright 2018, The Authors. Published by Optics Letters.

Additionally, Wang et al.^[^ ^64^ ^]^ utilized fsLdW to process light‐induced polymers with ion‐exchange properties, such as poly(2‐acrylamido‐2‐methyl‐1‐propanesulfonic acid) (PAMPS), to form negatively charged micro/nano‐structures in aqueous solutions. These structures can adsorb positively charged metal ions, nanoparticles, proteins, and other similar substances via electrostatic interactions. Moreover, PAMPS can be modified with ethylenediamine to produce positively charged micro/nano‐structures (**Figure** **13**). Hence, all charged materials can be functionalized with PAMPS micro/nano‐structures, offering new possibilities for the manipulation of charged particles.

#### 3.1.3 Embedded Components in Microfluidic Chip Devices

**Figure 14:**
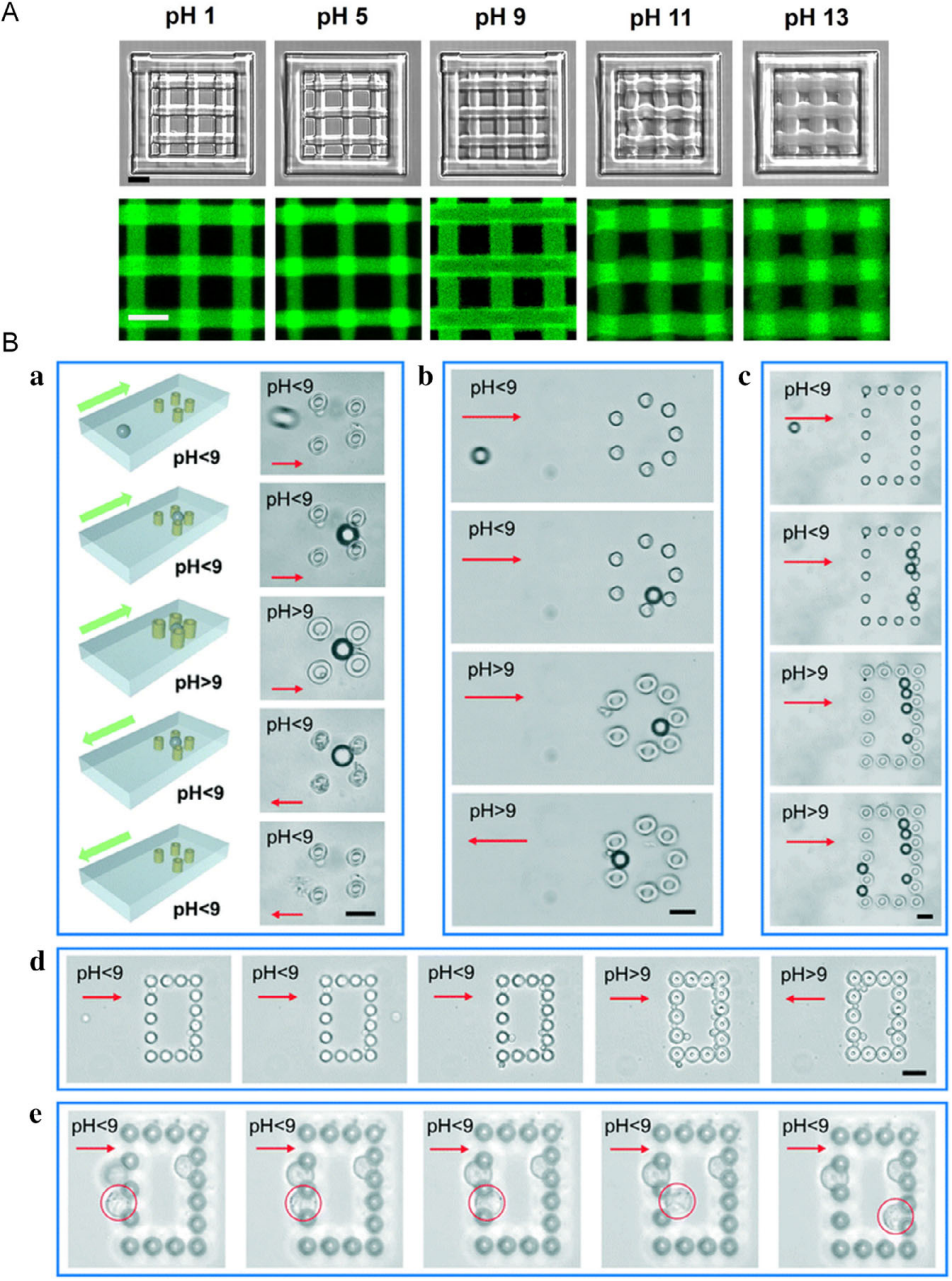
A) pH‐responsive mesh‐like structure expands when pH increasing from 1 to 13. Reproduced with permission.^[^ ^65^ ^]^ Copyright 2017, ACS. B) Procedure for complete trapping of the particle by changing the pH. (a) Schematic illustration and bright‐field microscopy images of the trapping and releasing procedure of the particle by tunable microfluidic device (TMFDs) with a trapezoidal‐trap array; (b,c) bright‐field microscopy images of the trapping procedure of the particle by TMFDs with a circular‐trap array and rectangle‐trap array, respectively; (d) bright‐field microscopy images of the trapping procedure of the yeast cells; (e) bright‐field microscopy images of the deformation of the neural stem cells that are bigger than the array gap. All scale bars: 20 μm. Reproduced with permission.^[^ ^1^ ^]^ Copyright 2019, RSC.

Embedding stimulus‐responsive hydrogel microactuators in microfluidic devices can enhance their capabilities for targeted microparticle screening and control. Wei et al.^[^ ^65^ ^]^ conducted a detailed study on the mass and 3D micro/nano‐structure processing capabilities of BSA structures fabricated via femtosecond‐laser‐induced TPP, achieving a lateral spatial resolution of up to 126 nm to ensure processing precision. Their research revealed that by altering the BSA concentration, the properties of the micro/nano‐structures can be controlled, including the surface morphology and swelling‐ratio variations, at different pH levels ranging from 1.08 to 2.71. Leveraging this discovery, they designed and manufactured specialized micro/nano‐structures mimicking the facial features of a panda with environmental responsiveness, showing reversible expression changes in deformation characteristics. They also designed and produced a mesh‐like micro/nano‐structure (**Figure** **14**A) in which the pore size could be adjusted by varying the pH. This holds significant application value for achieving microparticle separation and screening functionalities within devices embedded in microfluidic chips. Hu et al.^[^ ^1^ ^]^ developed a tunable microfluidic device by integrating pH‐sensitive hydrogel microring arrays into the microchannels. The transient reversible deformation of the microrings was completed in less than 200 ms. The reversible contraction and expansion of the microrings enabled the capture and release of small objects (Figure 14B). This device can be processed and integrated using femtosecond‐laser holographic processing methods without the need for precise external equipment support, thereby ensuring stable operation and offering broad application prospects in the field of biomedical analysis.

**Figure 15:**
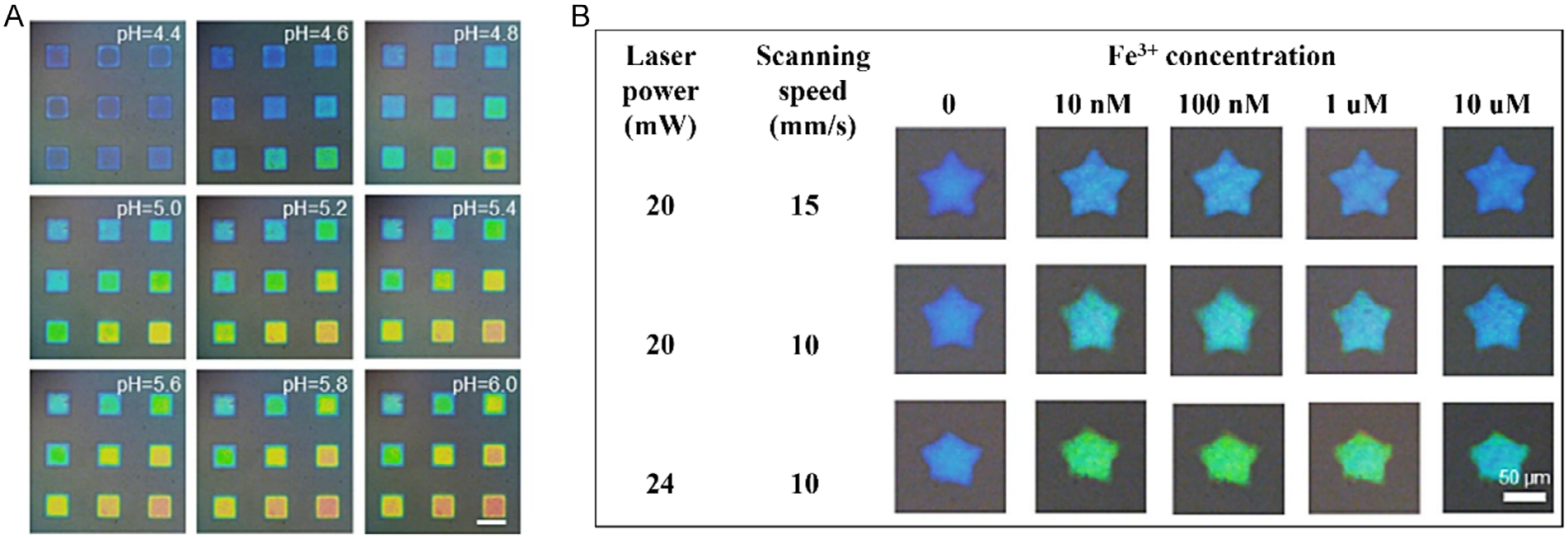
A) Optical images of the microsensor array under different pH. Scale bar: 100 μm. B) Photos of the microsensors processed under different parameters in solutions with different Fe^3+^ concentration. Reproduced with permission.^[^ ^66^ ^]^ Copyright 2024, Elsevier.

In addition, Embedded components in microfluidic chip devices can also play an important role in sensing. Photonic crystal hydrogels (PCHs) have emerged as a promising material for in situ sensing applications in microfluidic devices due to their unique ability to convert environmental stimuli into optical signals through structural color changes. Traditional PCH sensors, typically fabricated as thin films, spheres, or fibers, are often limited by manual assembly and poor spatial integration, restricting their functionality in microfluidic environments. Liu et al.^[^ ^66^ ^]^ addressed these limitations by developing a versatile fabrication strategy based on direct laser writing (DLW) using TPL. This method enables the precise construction of 3D microsensors directly onto target substrates, such as micropillar arrays or microchannel walls, overcoming challenges in geometry control and spatial positioning. The resulting sensors demonstrated tunable responsiveness to pH and metal ion concentration, with their performance easily adjusted via laser processing parameters. Moreover, Liu et al. showcased the spatiotemporal sensing capabilities of these PCH sensors, enabling real‐time monitoring of pH diffusion and ion gradients within dynamic microenvironments (**Figure** **15**). This breakthrough highlights the potential of DLW‐fabricated PCH systems for advanced applications in cell culture, drug screening, and environmental monitoring, where noninvasive and high‐resolution sensing is critical.

### 3.2 Drug Delivery

**Figure 16:**
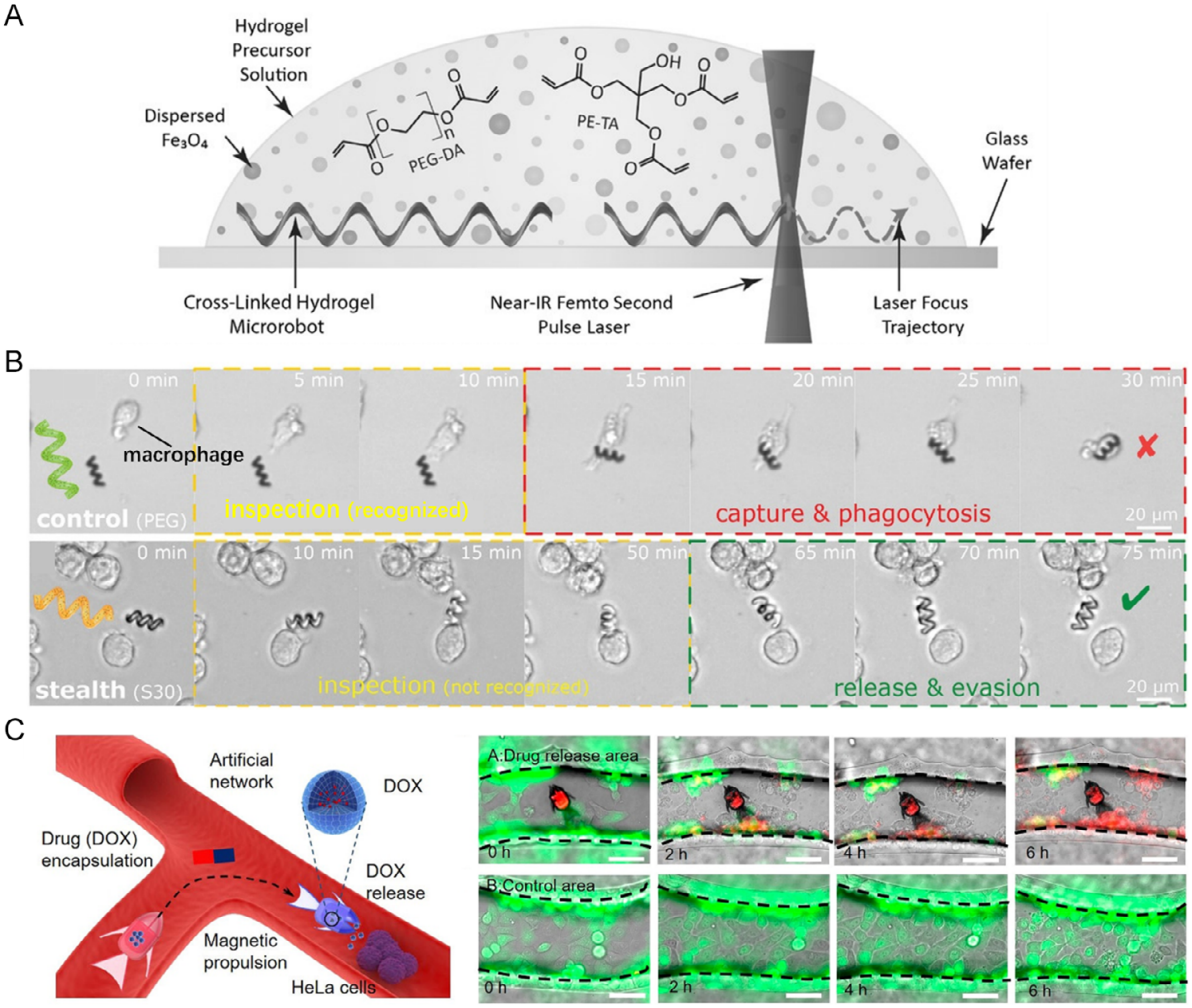
A) Laser direct‐writing fabrication of artificial bacterial flagellum microswimmers. Reproduced with permission.^[^ ^67^ ^]^ Copyright 2015, Wiley‐VCH. B) Comparison between traditional PEG microswimmers (above) and amphiphilic ionogel microswimmers (below) encountering macrophages. Reproduced under the terms of the CC‐BY 4.0 license.^[^ ^70^ ^]^ Copyright 2020, The Authors. Published by Advanced Materials. C) Process and efficacy of drug encapsulation, transportation, and release by microswimmers (red indicates low cancer cell activity, green indicates high activity). Reproduced with permission.^[^ ^71^ ^]^ Copyright 2021, ACS.

In precision medicine, targeted drug delivery within the human body is crucial; however, the precise delivery of drugs to lesion sites and their subsequent release remains a challenging task. Microswimmers fabricated from hydrogels are compact devices capable of controlled directional movement within the human body and hold promise for addressing this challenge. Peters et al.^[^ ^67^ ^]^ introduced an ultra‐superparamagnetic polymer material composed of iron‐oxide nanoparticles (Fe3O4), PEGDA, and tetraethylene glycol diacrylate to design a hydrogel with an artificial bacterial flagellum structure (**Figure** **16**A). Utilizing a weak rotating magnetic field to propel its helical motion wirelessly, they targeted drug transport to 3T3 fibroblast cells. The resulting degradation products exhibited low toxicity to cells, were degraded in physiological environments, and could be excreted, thereby providing convenient preparation, efficient propulsion, and new technical advantages for device applications in drug delivery. Wang et al.^[^ ^68^ ^]^ produced helical microswimmers from methacrylated gelatin (GelMA) hydrogels, which were noncytotoxic and biodegradable, by decorating their surfaces with magnetic nanoparticles to achieve magnetic responsiveness. These soft properties enabled them to maintain relatively high forward velocities as the rotation frequency increased, compared to previous rigid helical microswimmers. Hu et al.^[^ ^69^ ^]^ manufactured pH‐responsive hydrogel microcage structures capable of capturing and releasing microparticles by exploiting the differences in pore size during the expansion and contraction states. By coating the microcages with a magnetic layer, the captured particles could be controllably transported using magnetic‐field manipulation. However, when microswimmers engage in drug delivery within the human body, they must confront the immune defense mechanisms of the body against external threats. Cabanach et al.^[^ ^70^ ^]^ developed an amphiphilic ionogel and fabricated nonimmunogenic amphiphilic microswimmers that evaded recognition by immune cells. These amphiphilic materials could undergo extensive functionalization, such as the adjustment of mechanical properties, the incorporation of magnetic nanoparticles, the encapsulation of biomolecules, and surface functionalization. Moreover, these amphiphilic microswimmers remained undetected even after being tested for over 90 h by macrophages (Figure 16B), demonstrating their immunoevasive characteristics, which may establish a new technological foundation for preparing drug‐delivery devices in the biomedical field. Xin et al.^[^ ^71^ ^]^ adjusted the crosslinking density of pH‐responsive hydrogels by controlling laser parameters to fabricate environment‐adaptable deformable microactuators capable of grasping and releasing microparticles. By immersing these microactuators in a suspension of magnetic particles (Fe_3_O_4_ nanoparticles) for 12 h to induce magnetization, microswimmers with simultaneous grasping, transportation, and release functions were produced (Figure 16C). Xin et al.^[^ ^72^ ^]^ proposed two 4D‐printing strategies to manufacture deformable microswimmers based on pH‐responsive hydrogels, and their spatial shape‐changing capabilities could be utilized for navigating narrow micronetworks, offering significant potential for targeted drug transport within microcapillaries.

### 3.3 Microscaffolds

**Figure 17:**
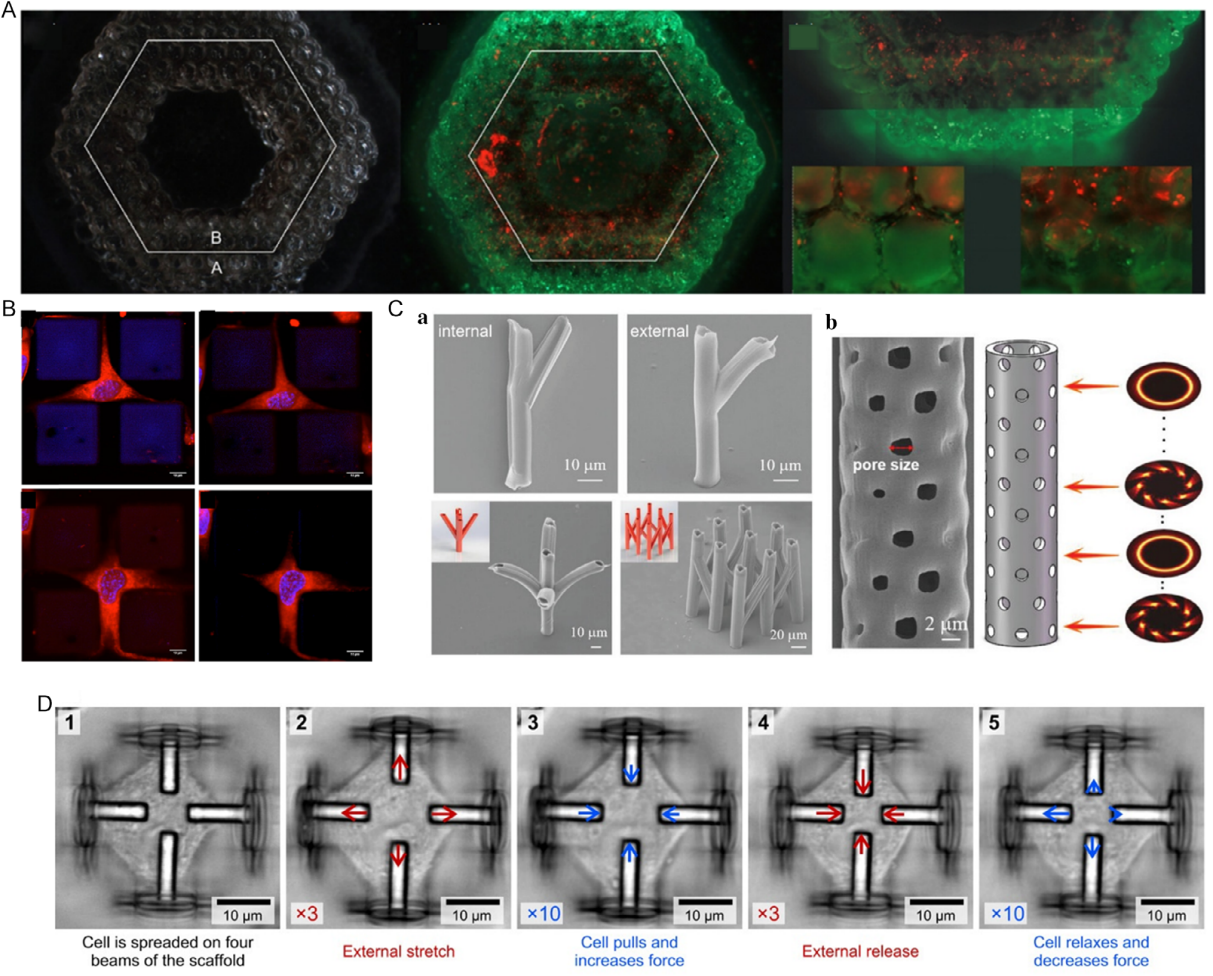
A) Dark‐field imaging of scaffold cocultured with cells using the LIFT method, fluorescence images of different cell types post‐LIFT, and detailed image of the boundary region. Reproduced with permission.^[^ ^73^ ^]^ Copyright 2010, IOP. B) Cultivation of NIH‐3T3 cells on a photoresponsive hydrogel scaffold, with images on the left showing the state before illumination and images on the right showing the state after illumination. Reproduced with permission.^[^ ^75^ ^]^ Copyright 2018, ACS. C) (a) Simple bifurcated microtubes and complex microtube networks, porous microtubes; (b) the processing method of biomimetic porous microtubes (right) and SEM images of porous microtubes with a diameter of 12 μm and a pore size of 2 μm (left). Reproduced with permission.^[^ ^78^ ^]^ Copyright 2023, Wiley‐VCH. D) Optical microscopic images at different time points during stretching experiments, with arrows indicating composite microscaffold beam movement triggered by external stimuli (in red) and cellular responses (in blue). Reproduced under the terms of the CC‐BY 4.0 license.^[^ ^77^ ^]^ Copyright 2019, The Authors. Published by Science Advances.

In cell engineering, the 3D microenvironment that hosts cells regulates and influences cell growth. 3D microscaffolds play a supportive role in the initial stages of tissue formation, where the scaffold porosity and internal pore structure impact cell migration, biodegradation kinetics, nutrient diffusion, and mechanical stability. Construction of 3D hydrogel microenvironments with predetermined geometric shapes and porosities is crucial for tissue engineering and regenerative medicine. To regulate cell‐growth behavior within scaffolds, control cell migration, and facilitate cell‐cell interactions, supportive 3D microscaffolds mimicking cellular‐growth environments are necessary. Therefore, the development of 3D hydrogel microscaffolds with properties similar to those of the ECM is a challenging issue that requires urgent attention. Femtosecond‐laser 3D processing, which is characterized by high precision, resolution, light‐utilization efficiency, and adjustable parameters, presents a robust technical support for tackling this challenge. Ovsianikov et al.^[^ ^73^ ^]^ utilized TPP technology to fabricate 3D micro/nano‐structures at the submicron scale using PEGDA with a molecular weight of 742 Da. They demonstrated the successful seeding of cells onto highly porous 3D cell scaffolds through laser‐induced forward transfer (LIFT) (**Figure** **17**A), showcasing the ability to print various cell types on 3D scaffolds. The highly porous nature of the PEG cell‐culture medium prepared ex vivo aids in transporting nutrients and removing metabolic waste while also promoting cell attachment, proliferation, and migration. The combined approach of LIFT and TPP offers a novel method for constructing 3D multicellular tissues at the microscale and artificially synthesizing the ECM. Yuan et al.^[^ ^74^ ^]^ investigated the biological characteristics and processability of GelMA hydrogel solutions and showed that photosensitive GelMA hydrogel solutions exhibited excellent biological and processing performances suitable for cell adhesion. The GelMA hydrogel microscaffolds printed using the laser direct‐writing TPP method demonstrated good biocompatibility. Pennacchio et al.^[^ ^75^ ^]^ designed and manufactured optically responsive hydrogel micro/nano‐structures containing azobenzene (Figure 17B) capable of reshaping based on the shape of NIH‐3T3 fibroblast cells. The inclusion of azobenzene crosslinkers significantly enhanced the reproducibility and structural stability of micrometer‐scale materials, demonstrating the possibility of light‐driven gelation and enabling precise single‐cell deformation in biocompatible environments with high spatial and temporal accuracy. These findings illustrate how such structures can serve as theoretical and experimental bases for real‐time research on multifunctional smart hydrogel microscaffolds and guide cell‐force transmission processes. Yu et al.^[^ ^76^ ^]^ prepared high‐precision 3D cell microscaffolds using a photoresist comprising 40 wt% PEGDA and 60 wt% PE‐3A, which offered a more cell‐like environment for cell spreading than other microscaffolds with varying PE‐3A contents. 3D hydrogel cell microscaffolds prepared using photoresists containing chitosan demonstrated an optimized biocompatibility of the photoresist. Hippler et al.^[^ ^77^ ^]^ proposed a novel stimulus‐responsive photoresist, wherein *β*‐cyclodextrin is the host and interacts with adamantane as the guest through noncovalent, directed interactions to form crosslinks. By combining this photoresist with traditional photoresists, composite scaffolds with protein‐repellent base structures, protein‐adhesive sites, and controllable stimulus‐responsive hydrogels were created. This scaffold enabled the precise stretching of individual cells for related studies at spatial and temporal scales (Figure 17D), facilitating the detailed examination of numerous individual cells within the microenvironment. Song et al.^[^ ^78^ ^]^ used holographic femtosecond‐laser technology to generate programmable, morphologically bifurcated microtubes and porous microtube structures via TPP (Figure 17C). Porous microtubes were employed as 3D capillary‐network scaffolds for cultivating human umbilical‐vein endothelial cells to facilitate nutrient and metabolite exchange. This flexible and rapid capillary‐network fabrication technique provides a convenient multifunctional platform for vascular physiology, tissue regeneration, and other biomedical applications. Schwegler et al.^[^ ^79^ ^]^ utilized fsLDW technology to manufacture hydrogel 3D micro/nano‐structures. Certain regions of the micro/nano‐structure were designed to promote cell adhesion, whereas other regions repelled cells. Specific peptides were incorporated into the hydrogel in the cell‐adhesive regions, whereas other regions lacked them. By employing fsLDW to combine different hydrogels, a composite microscaffold with both cell‐adhesive and cell‐repelling properties was formed. The incorporated peptides were fluorescently labeled, enabling the visualization of cell‐adhesive regions. This method can potentially be applied in cell research in complex environments. Qiu et al.^[^ ^80^ ^]^ developed a novel single‐pulse multiphoton polymerization (SP‐MPP) method, utilizing dual‐aperture optical modulation to achieve annular laser spot distribution, significantly enhancing axial processing efficiency by 1–2 orders of magnitude compared to traditional layer‐by‐layer scanning. Using SZ2080 photoresist, the process yielded microtubes and micropillars with heights up to 130 μm and aspect ratios of 16:1, with tunable geometries dictated by laser energy and substrate positioning. These microtube arrays demonstrated exceptional functionality in 3D neuronal culture, promoting neuron proliferation by approximately twofold, enhancing synaptic differentiation to form up to nine synapses per neuron, and enabling precise directional growth with an alignment error under ±7.5°. Furthermore, patterned microtube arrays facilitated large‐scale 3D neuronal orientation and network formation, offering a versatile platform for in vitro neural tissue modeling and regenerative medicine. This work underscores the transformative potential of femtosecond laser SP‐MPP technology in advancing biomimetic microenvironments for complex biological applications. Inspired by the pericellular matrix (PCM) structure of cartilage, Li et al.^[^ ^81^ ^]^ developed a novel femtosecond laser maskless optical projection lithography (FL‐MOPL) technique to fabricate 3D PCM‐like hydrogel scaffolds for maintaining chondrocyte phenotype and promoting hyaline cartilage regeneration. Using bovine serum albumin‐glyceryl methacrylate (BSA‐GMA) as the hydrogel precursor, the study achieved precise control over scaffold geometry, pore size, and stiffness, with Young's moduli ranging from 20 to 163 kPa. The PCM‐like scaffolds were designed in three sizes (S, M, L) to explore the effects of spatial confinement on chondrocyte behavior. Among these, the M‐sized scaffold provided optimal geometric constraints, preserving the rounded morphology of chondrocytes and preventing dedifferentiation or hypertrophy. The M scaffold also significantly enhanced the synthesis of ECM components such as type II collagen (Col II) and glycosaminoglycans (sGAG) while suppressing markers of fibrosis (Col I) and hypertrophy (Col X). These findings demonstrate that scaffold geometry and stiffness synergistically regulate mechanotransduction and cell fate. This work highlights the potential of FL‐MOPL‐fabricated PCM‐like scaffolds for applications in cartilage tissue engineering, offering an efficient, high‐precision method to create biomimetic microenvironments for cell culture and regenerative medicine. Nakielski et al.^[^ ^82^ ^]^ presents an innovative approach to intervertebral disc (IVD) regeneration by developing injectable PLGA microscaffolds with enhanced microporosity induced by femtosecond laser structuration, specifically designed for the delivery of human nucleus pulposus cells (hNPCs). These microscaffolds, fabricated via electrospinning and laser processing at 1030 nm wavelength with precise pulse durations and energies, demonstrated improved cell penetration and colonization. Gamma sterilization was effectively utilized for terminal sterilization, causing minimal polymer degradation while preserving scaffold integrity. In vitro assessments confirmed the biocompatibility and cytocompatibility of the microscaffolds, which supported hNPC adhesion, proliferation, and survival. The microscaffolds also exhibited favorable injectability, potentially protecting cells from injection‐induced stresses, and promoted uniform cell distribution and proliferation within hydrogel constructs. This study underscores the potential of these laser‐structured, gamma‐sterilized PLGA microscaffolds in advancing regenerative medicine strategies for IVD degeneration and highlights the need for further optimization and in vivo evaluation. Li et al.^[^ ^83^ ^]^ developed a biocompatible bovine serum albumin‐glycidyl methacrylate (BSA‐GMA) hydrogel scaffold fabricated via FL‐MOPL, achieving high‐resolution and large‐area micro/nano‐structures for cartilage tissue engineering. The BSA‐GMA hydrogel, with a methacrylation degree of 65%, demonstrated controlled swelling (8.65% equilibrium swelling in PBS), enzymatic degradability (92% degradation over 7 days in collagenase), and excellent biocompatibility (93.11% relative cell growth rate after 7 days). Five distinct scaffold geometries including triangular, quadrilateral, pentagonal, hexagonal, and circular were designed to evaluate their effects on chondrocyte morphology. Circular scaffolds (*R*) with larger interior angles effectively maintained chondrocyte roundness and suppressed dedifferentiation, while triangular scaffolds (*T*) induced greater cell spreading and polygonal morphologies. The results underscore the significance of scaffold geometry in regulating chondrocyte behavior and highlight the FL‐MOPL technique as a versatile and precise tool for fabricating biofunctional hydrogels for cartilage repair and regeneration.

**Figure 18:**
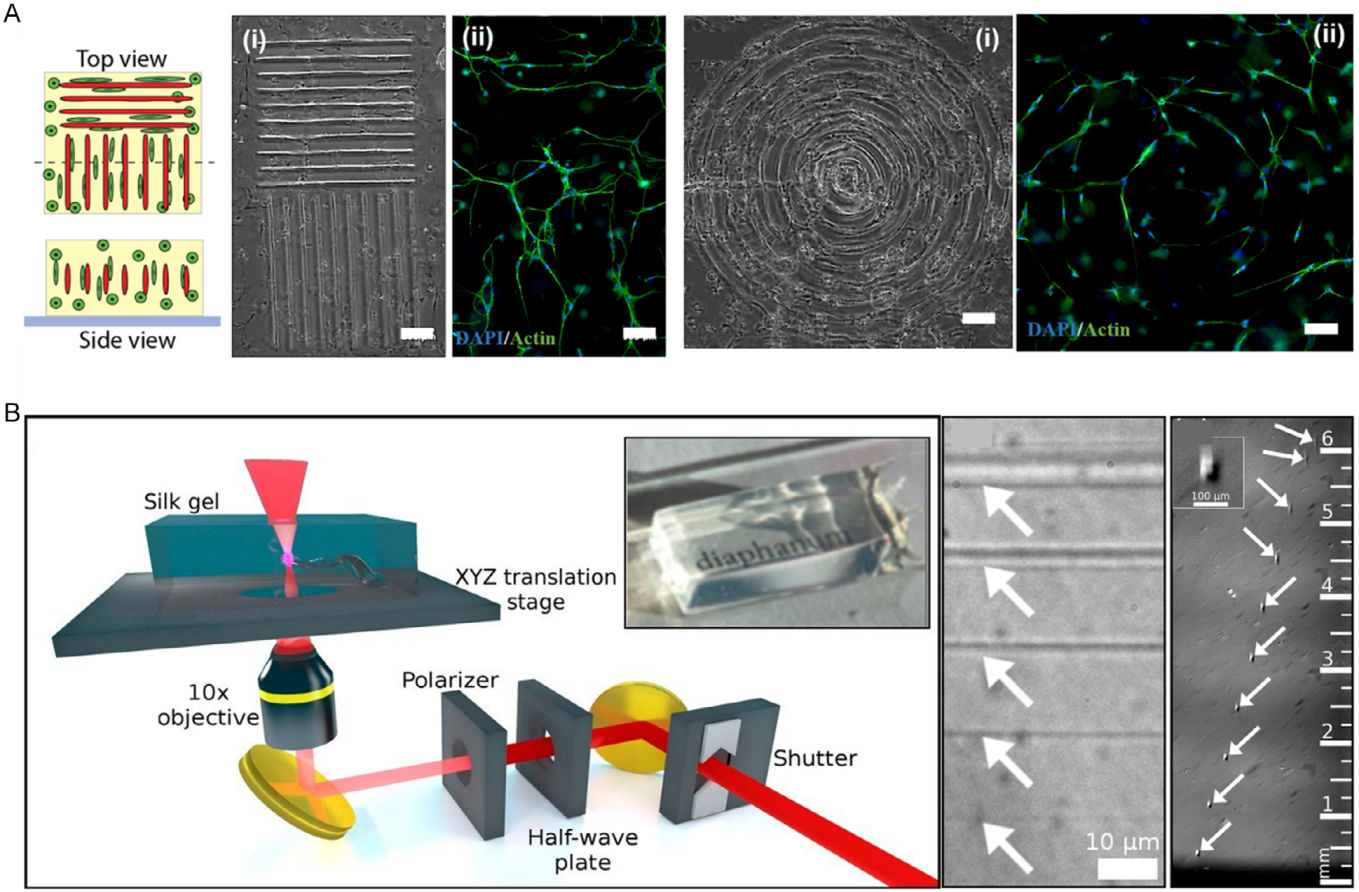
A) Cells preferentially align locally in the direction of dense lines as parallel lines and concentric circles are shown. Reproduced with permission.^[^ ^84^ ^]^ Copyright 2019, IOP. B) Schematic of microchannel patterning and images of microchannels fabricated within silk‐fibroin hydrogels. Reproduced under the terms of the CC‐BY 4.0 license.^[^ ^85^ ^]^ Copyright 2015, The Authors. Published by Proceedings of the National Academy of Sciences of the United States of America.

In addition to conventional 3D microscaffolds, densification patterns within hydrogel structures play a regulatory role in cell behavior. Xiong et al.^[^ ^84^ ^]^ utilized fsLDW technology to inscribe dense lines with widths of 1–5 μm within GelMA structures. The experiment revealed that these dense microlines led to the preferential local alignment of encapsulated human endothelial cells within GelMA (**Figure** **18**A), a phenomenon applicable to various cell lines with the potential for further expansion and development. The generation of microchannels or surface micropatterns in hydrogels through high‐power femtosecond‐laser ablation has similar functions. Applegate et al.^[^ ^85^ ^]^ employed moderate‐energy femtosecond lasers to create 2D and 3D multiscale microchannel patterns within silk‐fibroin hydrogels (Figure 18B), a process that is feasible for cell‐laden hydrogels without harming live cells. The resulting feature structures could guide cells in 3D, enhance cell infiltration within the hydrogels, and maintain pattern integrity. Hribar et al.^[^ ^86^ ^]^ used gold nanorods embedded in cell‐laden collagen hydrogels, focusing on near‐infrared femtosecond laser beams for patterned microchannel fabrication, facilitating cell migration, proliferation, and 3D alignment within the channels. Park et al.^[^ ^87^ ^]^ patterned graphene oxide/polyacrylamide (GO/PAAm) hydrogels using femtosecond‐laser ablation and chemical reduction to create a conductive, soft, and adaptive terrain environment. Through in vitro studies on C2C12 myoblasts, patterned hydrogels proved superior in inducing myogenesis and myotube alignment compared with unpatterned matrices. These hydrogels serve as versatile biomaterials with morphological features conducive to cell growth, mechanical properties similar to those of natural skeletal‐muscle tissues, and ideal conductivity for transmitting electrical signals to cells, thereby finding application in various biomedical fields. Gehre et al.^[^ ^88^ ^]^ utilized a low‐power femtosecond laser to fabricate microchannel grids in hydrogels, successfully guiding the self‐organization of individual bone cells into a bone‐cell network at micrometer‐scale resolution. This technology holds promise for the ex vivo generation of bone‐cell networks of specified morphology in the future, offering immense application potential in fundamental skeletal research and translational skeletal studies.

Recent advancements in microscaffold manufacturing technologies are also noteworthy. Morozov et al.^[^ ^89^ ^]^ developed a novel method for fabricating thermoresponsive biofunctional hydrogel microstructures using maskless multiphoton photocrosslinking. This technique enables simultaneous photocrosslinking and covalent attachment of polymer chains onto solid substrates, resulting in well‐defined polymer networks with spatial control over their composition and functionality. Characterization via atomic force microscopy and surface plasmon resonance imaging revealed tunable mechanical properties (Young's modulus increasing from 19 to 30 kPa with temperature) and a quasiperiodic wrinkle‐pattern topology. Furthermore, the inclusion of methacrylic acid (MAA) units enabled postmodification with biomolecules such as antibodies, showcasing the potential for creating customizable biointerfaces. This method offers significant advantages over conventional UV‐crosslinking techniques, including improved biocompatibility and higher spatial precision, and holds promise for applications in tissue engineering, biosensing, and biomedical microdevices. He and He^[^ ^90^ ^]^ demonstrated the fabrication of high‐resolution 3D micro‐nano hydrogel scaffolds using TPP with a custom femtosecond laser system operating at 780 nm. Gelatin methacryloyl (GelMA) hydrogel was utilized as the primary material due to its excellent biocompatibility and tunable properties, with optimal photoinitiator concentrations determined to be 0.3–0.5% w/v. The TPP process achieved a minimum resolution of 200 nm under a 20× objective lens (NA = 0.4) at a laser power of 2.2 mW and a scanning speed of 90 μm s^−1^. The study successfully fabricated simple cuboid scaffolds with grid dimensions of 5.2 μm and thin wall thicknesses of 500 nm, as well as complex vascular‐like structures mimicking mouse neurovascular environments. Neural stem cells cultured on the 3D scaffolds exhibited enhanced cell adhesion, proliferation, and differentiation, with a twofold increase in cellular activity compared to flat GelMA substrates. This work highlights the potential of TPP‐fabricated hydrogel scaffolds for replicating in vivo environments in vitro, offering cost‐effective, high‐precision solutions for tissue engineering, regenerative medicine, and large‐scale cell culture applications.

### 3.4 In Vitro Simulation of Vascular Networks

**Figure 19:**
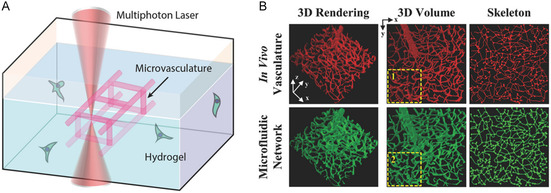
A) Microchannel‐network fabrication within hydrogels. Reproduced with permission.^[^ ^35^ ^]^ Copyright 2017, Wiley‐VCH. B) Confocal image stack of red cerebral cortex vessel network, with green representing the microchannel network fabricated within hydrogels based on the confocal image stack. Reproduced with permission.^[^ ^34^ ^]^ Copyright 2016, Wiley‐VCH.

The low‐power femtosecond‐laser irradiation of hydrogels can induce TPP, thereby enabling the fabrication of various 3D micro/nano‐structures. When high‐power fsLDW is applied to hydrogels, laser ablation leads to photodegradation within the hydrogel, enabling the creation of high‐resolution complex 3D microchannel structures inside the hydrogel. Utilizing these microchannel networks enables the in vitro simulation of biological vascular networks and other tissues, which is of significant importance in biomedical research. Heintz et al.^[^ ^34^ ^]^ utilized femtosecond‐laser technology to fabricate intricate, highly tortuous, and dense 3D biomimetic microchannel networks within PEGDA hydrogels. They demonstrated that microfluidic systems fabricated according to human vascular structures accurately replicated the structure, size, and density of microvessels in vivo. Additionally, they established two independent, yet closely interwoven, synthetic microvascular networks capable of interconnected transport over short distances, resembling in vivo vascular structures, potentially opening new avenues for constructing various extracorporeal vascular and quasi‐vascular tissues. Arakawa et al.^[^ ^35^ ^]^ implemented photodegradation processing in a synthetic peptide‐polymer‐based hydrogel material, creating perfusable vascular networks within the photosensitive gel. The vascular network dimensions span nearly physiological sizes, facilitating hierarchical tissue fabrication with full 4D control and enabling modifications to the processed vascular network over time based on user requirements (**Figure** **19**). Takayama et al.^[^ ^36^ ^]^ employed laser direct‐writing‐induced hydrogel degradation in PEGDA to manufacture hollow microchannels surrounded by gold‐nanoparticle ions. Given the excellent biocompatibility of hydrogels and gold nanoparticles, this technique holds promise for applications in tissue engineering, drug screening, and microfluidic chips.

## 4 Summary Table

**Table:** 

Processing principles	Functional structures of hydrogels	Functional characteristics	Materials for hydrogel production	Laser wavelength	Laser pulse width	Laser frequency	Applications and potential uses	References
Additive manufacturing	Microactuator structures	Ion‐concentration responsive	Aam, AMPS	780 nm	80 fs	82 MHz	Significant application potential in the biomedical field, offering vast opportunities in the fields of microactuators and micromanipulators.	[48]
		pH responsive and definition of shape change	BSA	780 nm	–	–	The ability to manipulate electromagnetic waves in the near‐infrared spectrum to control fluid dynamics is pivotal in both photonics and microfluidics; New avenues for the development of 3D reconfigurable crystals in the future.	[49]
		Humidity responsive	PEGDA	–	–	–	Functionalities such as data encryption, storage, and secure transmission; Broader applications in areas such as sensor systems, actuators, and soft‐robotics design.	[50]
		Temperature responsive	pNIPAM	–	–	–	Smart responsive structures.	[51]
		Photoresponsive	NIPAM	780 nm	120 fs	80 MHz		[52]
		Sugar responsive	Acrylamide; 3‐acrylamidophenylboronic acid; N,N’‐methylenebisacrylamide; tetrabutylphosphonium chloride; 7‐diethylamino‐3‐thenoylcoumarin	780 nm	120 fs	80 MHz	Continuous blood‐glucose monitoring; Composite material microgrippers; Crucial concepts for establishing responsive microrobotic structures capable of continuous and reversible actuation.	[42]
		Programmable, light‐driven intelligent microactuators	NIPAM; MBA; SWNTs	780 nm	120 fs	80 MHz	Biomedicine; Tissue engineering; Soft robotics.	[53]
		Programmable, biocompatible, self‐healing reconfiguration	NIPAM; MBA; TPO; PVP; Rhodamine 6 G	800 nm	75 fs	80 MHz	Hydrogel‐based microstructures in chiral photonics, optical sensing, and microfluidic systems.	[54]
		High‐freedom, programmable shape reconfiguration	NIPAM; PEG‐DA; TAIC; MB	800 nm	< 75 fs	–	Biomedicine; Microfluidics; Adaptive photonics	[55]
	Biomimetic structures	pH‐Driven Muscle‐Skeletal Composite Structure	BSA	800 nm	120 fs	80 MHz	Bio‐driven; Multi‐material 3D micro‐robot fabrication.	[56]
		Thermal‐driven humanoid joint	pNIPAM	–	–	–	Ability to construct micro‐robot systems with multiple programmable modes with the potential to achieve advanced applications in fields such as healthcare, biotechnology, and beyond.	[57]
		Reconfigurable microbot collectives	pNIPAM	–	–	–	Reconfigurable microbot collectives with dual magnetic and photothermal actuation; Adaptive cargo transport and targeted drug delivery.	[58]
	Microparticle manipulation	pH‐responsive 3D microtraps	BSA	780 nm	100–200 fs	–	Micro‐object capture through pH Modulation	[59]
		pH‐responsive Microactuator	pAAC	800 nm	75 fs	80 MHz	Manipulation of micro‐objects; Drug delivery, Vast prospects in various fields such as precision sensors and biomedical devices.	[60]
		pH‐driven deformable janus microcolumns	Acrylic acid; N‐isopropylacrylamide, polyvinylpyrrolidone; ethyl lactate; dipentaerythritol hexaacrylate; triethanolamine; 4,4′bis(diethylamino) benzophenone/N,N‐dimethylformamide solution	800 nm	75 fs	80 MHz	Smart display; Smart sensors, manipulation; Filtration, and transport of micro‐objects.	[61]
		pH‐responsive asymmetric microactuator	DMAEMA; PETA; PEGda; TPO; HMPP; Glycerol	780 nm	150 fs	100 MHz	Accurate capture and release of micro‐objects; Guidance for preparing smart hydrogel devices; Potential applications in research fields such as soft robotics, microsensors, and micro‐electro‐mechanical systems	[62]
		pH‐responsive microcage array	AAc; PETA; 4,4′‐bis(diethylamino) benzophenone (EMK)	–	–	–	The ability to observe cellular behavior within normal or altered biological settings and reveal the anticancer effects of drug‐loaded compounds serving as an array‐analysis platform; Potential for applications in targeted transport and biomedical analysis.	[63]
		pH and temperature co‐responsive microactuator	TPO; HMPP; PEGda; PETA; DMAEMA	780 nm	150 fs	100 MHz	Successfully accomplishing the dual‐stimulus cooperative response for manipulating micro‐objects; Potential applications in soft robotics, micro‐manipulation, among other fields.	[44]
		Ion‐exchange micro/nano‐structure	AMPS; PEGDA; RB	780 nm	120 fs	80 MHz	Manipulation of charged microparticles; Capable of printing functional 3D microvessels and producing novel biochips.	[64]
	Embedded components in microfluidic chips	pH‐responsive microgrid	BSA	780 nm	80 fs	80 MHz	Capable of sorting particles of varying sizes according to pH fluctuations; Potential applications in biomedicine and biosensors.	[65]
		pH‐responsive microfluidic integrated microring Array	NIPAAm; AAc; EL; DPEPA; TEA; EMK; DMF	800 nm	75 fs	80 MHz	Micro‐object manipulation; Single‐cell biology analysis	[1]
		Photonic crystal hydrogel sensors	Aam; AA; Bis; TPO	–	–	–	Online biochemical analysis	[66]
	Drug‐delivery devices	Magnetic‐driven artificial bacterial flagellar structure	PEGDA; PE‐TA; FF	–	–	–	Targeted drug delivery; Biomedical analysis	[67]
		Biodegradable helical microswimmer	Cyclopentanone; benzaldehyde 3‐[(4‐formyl‐phenyl)‐methyl‐amino]propionic acid	780 nm	–	80 MHz	Targeted drug delivery and capable of complete degradation by collagenase; Promoting cell adhesion and growth, being progressively digested by cells throughout the cultivation process; Has significant potential for applications in biomedicine.	[68]
		Magnetic‐driven pH‐responsive microcage	AAc; N‐isopropylacrylamide; PVP; ethyl lactate; dipentaerythritol hexaacrylate; triethanolamine; 4,4′bis(diethylamino) benzophenone/N,N‐dimethylformamide	800 nm	75 fs	80 MHz	Modulating pH to regulate drug capture and release; Drug delivery.	[69]
		Nonimmunogenic zwitterionic microswimmer	Carboxybetaine Methacrylate; Sulfobetaine Methacrylate; Carboxybetaine Dimethacrylate; Sulfobetaine Dimethacrylate	–	–	–	Possesses immune‐evasion properties; Introducing novel methods for fabricating drug‐delivery devices in the field of biomedicine.	[70]
		pH‐responsive deformable microswimmer	NIPAAm; AAc; PVP; EL; DPEHA; TEA; EMK; DMF	800 nm	75 fs	80 MHz	Application potential in terms of biopharmaceutical transportation	[71]
			NIPAAm; AAc; PVP; EL; DPEHA; TEA; EMK; DMF	800 nm	75 fs	80 MHz	The capability for spatial shape transformation can be harnessed to navigate through narrow micro‐networks and presents significant potential for targeted drug delivery within microcapillaries.	[72]
	Microscaffolds	Customized 3D microscaffold	PEGda	780 nm	120 fs	80 MHz	Micro‐scaffold 3D structures can be autonomously defined, allowing for precise control of cell density and positioning, holding significant importance for numerous tissue‐engineering applications and systematic in vitro studies on cell‐cell and cell‐matrix interactions.	[73]
		Photoresponsive microscaffold	GelMA	780 nm	–	–	The exemplary biological and processing characteristics of photosensitive GelMA hydrogel solutions have been established, showcasing their suitability for cell adhesion. These findings indicate broad prospects for applications in the field of tissue engineering.	[74]
		Photoresponsive Smart microscaffold	Acrylamide‐modified gelatin B	780 nm	100 fs	80 MHz	Real‐time investigation and guidance of cell force‐transduction processes; Facilitates the microfabrication of intricate 3D light‐responsive hydrogel structures to create a “dynamic” engineering platform and marks a shift from static smart materials employed in cell‐culture applications toward dynamic intelligent materials.	[75]
		Highly biocompatible microscaffold	PEGDA; PE‐3 A; SH; Chitosan	780 nm	–	–	Confirming that chitosan can optimize the biocompatibility of photoresists; Providing a flexible approach for manufacturing biocompatible 3D hydrogel cell scaffolds.	[76]
		Stimuli‐responsive composite microscaffold	Irgacure 819; PETA; βCD‐Aam, AdAAm	–	–	–	A composite scaffold has been engineered, featuring a protein‐repelling foundational structure, protein adhesion sites, and controllable stimulus‐responsive hydrogels. This scaffold enables the precise stretching of individual cells at spatial and temporal scales for pertinent research purposes. Moreover, it offers the ability to scale up investigations involving a multitude of individual cells within the microenvironment.	[77]
			PEGDA; Arg‐Gly‐Asp (RGD) peptide	780 nm	–	–	Cell research in complex environments	[78]
		Biomimetic branched microtubes and porous microtube scaffold	zirconium–silicon hybrid sol–gel material; 4,4′‐bis(diethylamino)‐benzophenone	800 nm	75 fs	80 MHz	Offering a convenient and versatile experimental platform for vascular physiology, tissue regeneration, and various other biomedical disciplines.	[79]
		Microscaffold with high‐aspect‐ratio microstructures	SZ2080	1030 nm	217 fs	200 kHz	Promoting neuron proliferation; Enhancing synaptic differentiation; Facilitating large‐scale 3D neuronal orientation and network formation	[80]
		Pericellular‐matrix‐like microscaffold	BSA‐GMA	532 nm	–	–	Creating biomimetic microenvironments for cell culture and regenerative medicine	[81]
		Microscaffold with enhanced microporosity	PLGA	1030 nm	270 fs	20/10 kHz	Delivery of human nucleus pulposus cells	[82]
		Patterned cartilage microscaffold	BSA‐GMA	400 nm	–	–	Regulating chondrocyte behavior and chondrocyte morphology; Cartilage repair and regeneration	[83]
Subtractive Manufacturing		Patterned hydrogel microscaffold	GelMA	730 nm	–	80 MHz	Inducing cells to arrange preferentially in localized patterns.	[84]
			Silk fibroin	810 nm	100 fs	80 MHz	Guiding cell growth to facilitate cell infiltration within the hydrogel while maintaining pattern integrity.	[85]
			VWR	800 nm	100 fs	80 MHz	Facilitating cell migration, proliferation, and 3D alignment within patterned channels.	[86]
			GO/PAAm	1030 nm	500 fs	–	A conductive, soft, and environmentally adaptive hydrogel has been engineered as a potent multifunctional biomaterial. It showcases morphology traits favorable for cell proliferation, mechanical attributes akin to natural skeletal muscle tissue, and optimal conductivity for facilitating electrical‐signal transmission to cells, thereby finding versatile applications across multiple biomedical domains.	[87]
		Microchannel grids in hydrogels	GelMA	780 nm	< 80 fs	–	Fundamental skeletal research; translational skeletal studies	[88]
		Thermoresponsive biofunctional hydrogel microstructure fabrication	pNIPAAm; BPAm; AAHAQ; MAA	785 nm	100 fs	80 MHz	Providing a new tool for designing controllable biointerfaces and responsive microstructures; Enabling the fabrication of more precise multifunctional 3D micro‐ and nanostructures	[89]
		Fabrication of high‐resolution 3D micro‐nano hydrogel microscaffolds	GelMA	780 nm	120 fs	100 MHz	Enhancing cell adhesion, proliferation, and differentiation; Increasing cellular activity; Offering cost‐effective, high‐precision solutions for tissue engineering, regenerative medicine, and large‐scale cell culture applications	[90]
	Microchannel networks	In vitro simulated vascular network	PEGDA	790 nm	140 fs	–	When conducting an in vitro simulation of human vascular networks, the two constructed microvascular networks can intricately intertwine while maintaining independence, bearing a striking resemblance to real vascular networks within the human body; The potential of novel avenues for building vascular and vascular‐like tissues for a range of in vitro applications.	[34]
			PEGtetraBCN; diazide N3‐oNB‐RGPQGIWGQGRGDSGK(N3)‐NH2 peptide	740 nm	–	80 MHz	The creation of a complete microchannel network resembling the natural human vascular system within hydrogels presents substantial potential for application in the fields of microfluidics and cell culture.	[35]
			PEGDA	800 nm	100 fs	1 kHz	The creation of hollow microchannels encompassed by gold nanofilaments within hydrogels exhibits promising application potential in tissue engineering, drug screening, and microfluidic chip technologies.	[36]

## 5 Conclusions and Outlook

This review focuses on the nonlinear optical properties of femtosecond lasers and the biological characteristics of hydrogel materials. It elaborates on the additive and subtractive fabrication methods of femtosecond‐laser interaction with the micro/nano‐structures of hydrogels, modification mechanisms of photon–electron‐ionization and multiphoton polymerization, and biomedical applications driven by structural morphology and functionality. By modulating parameters such as the pore size, distribution, shape, and crosslinking form of hydrogel micro/nano‐structures, femtosecond lasers induce distinct structural mechanical states and surface cell features, thereby enabling interventions in cellular processes such as proliferation, differentiation, deposition, migration, evaluation of drug efficacy in engineered tissues, and directional control over nutrient absorption and waste excretion. The introduction of femtosecond‐laser fabrication techniques optimizes the physical and chemical properties of ECM micro/nano‐structures based on hydrogels; their response modes and efficiency to external stimuli; and the impact of structural characteristics on cell behavior, signal transduction, biocompatibility, and other biological properties. This enhances the application potential of hydrogels in the biomedical field such as microrobotics, passive sensors, wound dressings, biophotonic waveguides, and flexible electronic devices.

Hydrogels are a kind of biocompatible polymers, which can be classified into two categories: biodegradable and nonbiodegradable ones. Biodegradable hydrogel polymers can degrade into nontoxic byproducts in biomedical applications. In fact, the use of biodegradable polymers with a low environmental footprint may be limited. Because when they are incorporated into the fabrication of micro/nano‐structures based on femtosecond lasers, they face challenges such as the stability of chemical bonds being easily affected by environmental factors, sensitivity during processing, and negative effects caused by degradation products. In response to these challenges, monomer polymerization can be precisely controlled by femtosecond laser pulses, laser parameters can be optimized, and the negative effects of degradation products can be suppressed, so as to effectively address these challenges.

However, some challenges and difficulties in the preparation of hydrogel micro/nano‐structures using a femtosecond laser still remain. 1) The complex manufacturing process and low production efficiency of femtosecond lasers result in high manufacturing costs. 2) The insufficient precision and consistency of femtosecond‐laser‐prepared hydrogel micro/nano‐structures severely affect the production stability of biomaterials. 3) The introduction of singular functionality results in the wastage of medical resources. 4) Inadequate biocompatibility reduces acceptability within organisms, thereby limiting scenarios for medical applications. 5) The lack of structural intelligence makes precise targeting difficult and hinders the advancement of smart‐healthcare systems. Thus, a hydrogel micro/nano‐structure preparation technology that is highly efficient, precise, functionally integrated, biocompatible, and intelligent must be developed. 6) Each preparation method has its own limitations. For instance, additive manufacturing exhibits constraints in both precision and efficiency when constructing complex structures. While subtractive manufacturing offers high precision, its overall efficiency remains low, and it lacks sufficient flexibility in 3D construction. Although 3D printing is effective in macroforming, it faces challenges in achieving adequate precision and functionalization at the microscale.

To address these challenges and difficulties, we propose several improvement methods. 1) By combining the laser‐induced tunnel ionization theory and femtosecond‐laser field‐distribution control mechanisms, a multidimensional energy‐field transformation coupling model can be established. This should be followed by the optimization of manufacturing‐equipment processes, simplification of preparation processes, and enhancement of the preparation efficiency of the hydrogel micro/nano‐structures, thereby reducing manufacturing costs. 2) We introduce real‐time monitoring and feedback systems tailored for the femtosecond‐laser preparation process and autonomously develop iterative optimization algorithms to ensure the precision and uniformity of each micro/nano‐structure, thereby enhancing the production stability. 3) Utilizing hydrodynamic material models, tensile‐stress cutoff negative‐pressure effects, and transient and dynamic load theories, the diverse optical‐field‐shaping capabilities of femtosecond lasers can be combined with the stimulus‐response mechanism of hydrogels to achieve the rapid joining and formation of complex functional integrated structures, thereby increasing the utilization of medical resources. 4) A relationship model between the femtosecond‐laser action and hydrogel‐biocompatibility evaluation should be established. The correlation curves between preparation processes and compatibility results should be analyzed and predicted, preparation technology schemes can be adjusted, and material formulations should be optimized based on different application scenarios to enhance acceptability in biomedical applications and broaden applications in the biomedical field. 5) Through intelligent structural design and preparation, micro/nano‐structures can self‐regulate and drive according to specific microenvironments, thereby achieving automatic recognition, adhesion, proliferation, and treatment functions of hydrogel micro/nano‐structures for targeted delivery positions and driving the rapid development of smart‐healthcare technologies. 6) The application of hybrid technologies combines the advantages of both additive and subtractive manufacturing. Additive manufacturing enables rapid construction of structures but may suffer from structural defects, while subtractive manufacturing offers high‐precision refinement. A rational combination of the two, considering process sequence and parameters, not only enhances processing efficiency but also improves product quality, meeting the stringent requirements for high‐precision, complex structures in the biomedical field. When integrated with 3D printing, the process begins with the construction of the macroscopic shape, followed by femtosecond‐laser processing to refine microscopic structures and introduce functional modifications. This approach compensates for the limitations of 3D printing at the microscale, expanding the potential applications of hydrogel structures in the biomedical sector and offering innovative concepts and methodologies for advancing this technology in both biomedical and related fields.

In the future development of biomedical sciences, femtosecond‐laser fabrication technology for hydrogel micro/nano‐structures will undergo further technological improvements and innovations, deepening and expanding biomedical applications, multifunctional design and customized preparation, interdisciplinary collaboration, industry–academia–research integration, and considerations of safety and sustainability. 1) By introducing real‐time adjustable beam‐focusing technology and process‐monitoring feedback and exploring new material combinations and fabrication processes, the innovation and practicality of fabrication technology can be enhanced, expanding its potential applications in the field of biomedicine. 2) As the understanding of the compatibility and stability of hydrogel micro/nano‐structures within organisms deepens, beyond traditional drug delivery and tissue engineering, exploration can extend to applications in biosensing, medical imaging, and gene delivery. For example, hydrogel micro/nano‐structures can be used as carriers to achieve intelligent targeted drug delivery or gene transfer, and they can be incorporated into biosensors for detecting biomarkers or monitoring physiological states. 3) Future studies will focus on designing and fabricating hydrogel micro/nano‐structures with multiple functions and customizing their preparation based on specific application requirements. For example, the design of hydrogel micro/nano‐structures with adjustable pore structures or surface functionalization can enhance biocompatibility in tissue engineering. Additionally, the customized preparation of suitable hydrogel micro/nano‐structures based on different disease characteristics or individual traits can facilitate personalized medical treatment plans. 4) Interdisciplinary cooperation and communication can be facilitated in fields such as materials science, optical engineering, and biomedical engineering. Collaboration with medical professionals, researchers, and pharmaceutical companies in the biomedical field will accelerate the translation of hydrogel micro/nano‐structures into clinical applications. Furthermore, industry–academia–research integration will drive the commercialization of the technology, accelerating its promotion and application in the market. 5) Future developments will place greater emphasis on the safety and sustainability of hydrogel micro/nano‐structures. During the design and fabrication processes, the biocompatibility, degradability, and environmental friendliness of materials must be considered to ensure their safety and sustainability in clinical applications and biological environments. Additionally, monitoring and evaluating the long‐term biocompatibility and stability of hydrogel micro/nano‐structures during extended use are essential to ensure their safe and effective application in the clinical and biomedical fields.

## Conflict of Interest

The authors declare no conflict of interest.

## Author Contributions

**Jingyun Ma**: conceptualization (lead); project administration (lead); writing—original draft (lead); writing—review editing (lead). **Jinchi Wu**: writing—review editing (equal); writing—original draft (equal); investigation (lead). **Zheli Lin**: data curation (lead); investigation (equal); writing—original draft (equal). **Jin Wang**: methodology (lead); supervision (equal). **Wenhao Yao**: conceptualization (lead); methodology (equal). **Yi Zhang**: conceptualization (equal); methodology (lead). **Xinquan Zhang**: methodology (lead); validation (equal). **Limin Zhu**: methodology (equal); validation (lead). **Yoshio Hayasaki**: supervision (lead). **Honghao Zhang**: conceptualization (lead); supervision (lead); writing—review editing (lead).
